# Recent progress in terahertz metamaterial modulators

**DOI:** 10.1515/nanoph-2021-0803

**Published:** 2022-04-11

**Authors:** Riccardo Degl’Innocenti, Hungyen Lin, Miguel Navarro-Cía

**Affiliations:** Department of Engineering, University of Lancaster, Bailrigigg, Lancaster LA1 4YW, UK; School of Physics and Astronomy, University of Birmingham, B15 2TT Birmingham, UK; Department of Electronic, Electrical and Systems Engineering, University of Birmingham, Birmingham B15 2TT, UK

**Keywords:** metamaterials, modulators, terahertz

## Abstract

The terahertz (0.1–10 THz) range represents a fast-evolving research and industrial field. The great interest for this portion of the electromagnetic spectrum, which lies between the photonics and the electronics ranges, stems from the unique and disruptive sectors where this radiation finds applications in, such as spectroscopy, quantum electronics, sensing and wireless communications beyond 5G. Engineering the propagation of terahertz light has always proved to be an intrinsically difficult task and for a long time it has been the bottleneck hindering the full exploitation of the terahertz spectrum. Amongst the different approaches that have been proposed so far for terahertz signal manipulation, the implementation of metamaterials has proved to be the most successful one, owing to the relative ease of realisation, high efficiency and spectral versatility. In this review, we present the latest developments in terahertz modulators based on metamaterials, while highlighting a few selected key applications in sensing, wireless communications and quantum electronics, which have particularly benefitted from these developments.

## 1 Introduction

**Figure 1:**
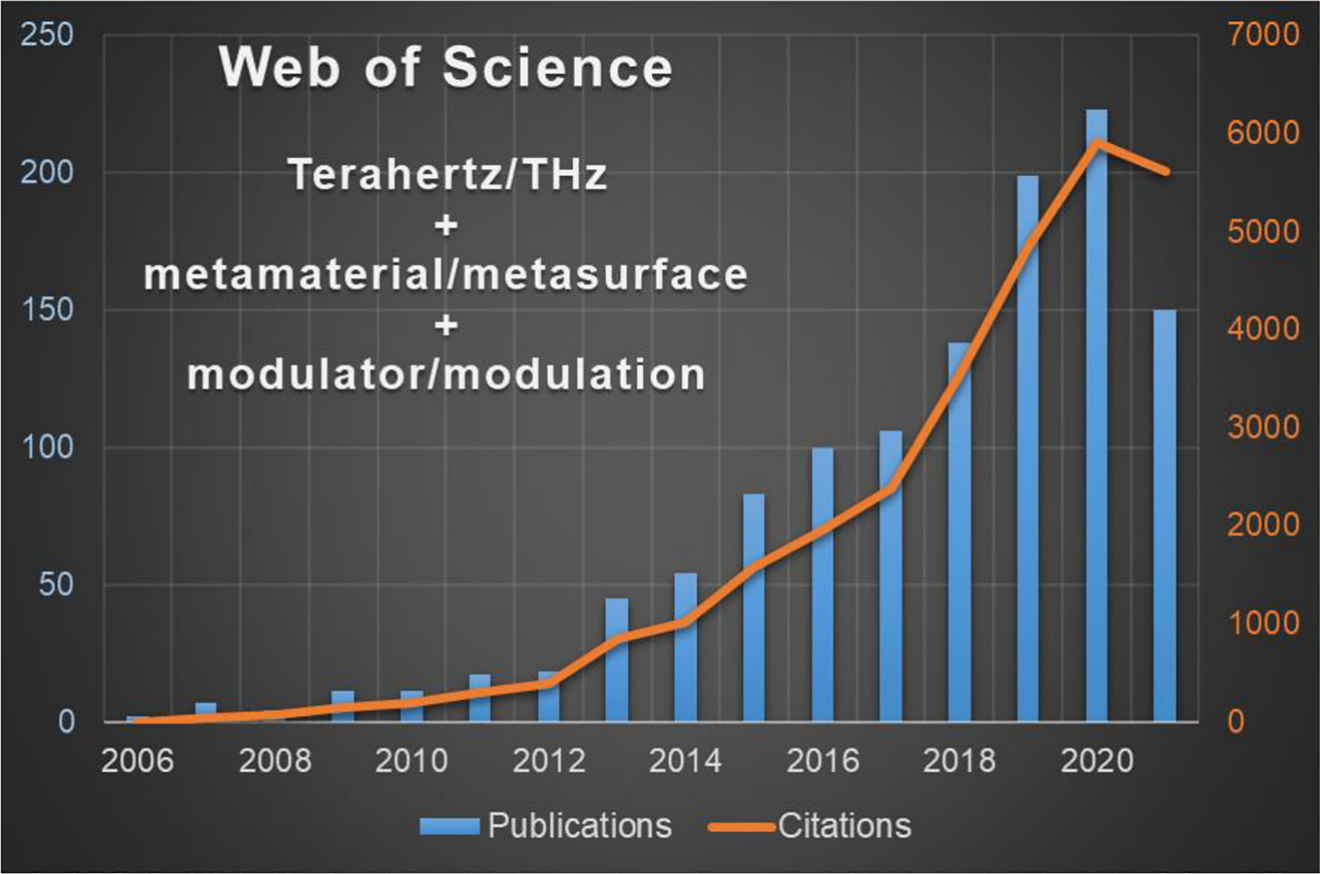
Historical evolution of publications on terahertz metamaterial modulators and the corresponding citation record (citation record at 09/12/2021).

The terahertz (THz) range is broadly defined as the portion of the electromagnetic spectrum spanning from 0.1 to 10 THz, corresponding to vacuum wavelengths from 3000 to 30 μm. This spectral region coincides with the length scale between electronics and photonics, which has seen a tremendous growth in the latest years [1], [2], [3], [4], [5]. This was initially made possible because of the great technological progresses in coherent compact sources such as the resonant tunnelling diodes (RTDs), uni-traveling carrier (UTC) photodiodes, quantum cascade lasers, time domain fs-laser spectrometers and detectors, e.g., Schottky photomixers. Now, it is driven by its great potential for, *in primis*, the next generation of wireless communications, imaging, spectroscopy and sensing [6], [7], [8], [9]. The potential of THz in these application domains arises from the large available bandwidth, the ability of THz radiation to penetrate through many optically opaque materials (e.g., clothing, paper, etc.), achievable spatial (sub-)millimetre resolution and the fact that specific rotations, vibrations or librations of molecules and molecular aggregates occur in this frequency range. Efficient manipulation of the THz radiation is however still lagging thus hindering the full exploitation of all these applications. The lack of a suitable optoelectronics circuitry for THz signal manipulation has driven an intense research effort recently. A few reviews [10], [11], [12], [13], [14], [15], [16], [17], [18], [19], [20], [21] have addressed this fast-evolving field centred on specific features, methodology or application sector. Here, we provide an overview of the latest results in this fascinating research area keeping a large view on the different materials and approaches used, focusing our attention to metamaterial-based methodologies [10], [11], [12], [13], [14], [15], [16], [17] and reporting the main results achieved in the latest ∼5 years, without dwelling on the fabrication methods, but rather highlighting the pertinent figures of merit (FOMs). Also, this review aims to target complex photonic approaches rather than a pure material research. Because of their growing implementation and diffusion in many research areas, this review only considers metamaterial-based devices, in their broader definition. Within the metamaterial paradigm and its two-dimensional equivalent metasurface, it is possible to widen the material design opportunities. This is accomplished by engineering the metamaterial’s subwavelength unit cell whereby the material properties of the composite material are controlled not only by varying the chemical composition as in natural materials, but also by the unit cell’s shape, internal structure, position and orientation. Extensive definitions of metamaterials and of their operating principles can be found in dedicated reviews [10], [11], [12], [13], [14], [15], [16], [17]. The metamaterial’s property engineering peculiarity, together with the strong light–matter interaction granted by the unit cell’s subwavelength nature, and the metamaterials’ extreme versatility make them the ideal candidate for the realization of THz modulators. A testament of this is the recent increasing attention by the scientific community, as showed in Figure 1. Finally, the metamaterials’ tuneable frequency response is the key for the achievement of a common agile platform capable of operating with minimal change in efficiency over the whole THz spectrum. Whilst the classical definition of metamaterials is normally used for addressing composites comprising split ring resonators or similar resonant particles, recent papers tend to use such a definition for describing complex photonic structures and the border between these concepts becomes blurred. Therefore, in order to adopt an inclusive point of view and to provide at the same time a comparison with other meaningful approaches, we decided to mention as well selected works whose non-conventional electromagnetic response and photonic emission could be consistent with metamaterials.

This review is structured as follows. In the first section, the main physical mechanisms at the base of modulations are reported, with an emphasis on the ones which are more likely to be used in applications beyond the laboratory environment. These include the all-optical and all-electronic modulation by using semiconductors, superconductors (SC), two dimensional (2D) materials, liquid crystals micro-electromechanical-systems (MEMS) and perovskites. In the following sections, amplitude, frequency and polarization modulators are reported. Finally, a few hot topic research areas will be reviewed, which are particularly profiting from the great progress in THz metamaterial modulators; namely quantum electronics, THz wireless communications, and sensing.

## 2 Basic operating principles and materials

Active modulation has been achieved in a variety of configurations and using several materials. We briefly report the more commonly used ones and their basic operating principles.

### 2.1 2D materials

2D materials have been an interesting functional material of choice for realising THz modulators because of their non-conventional characteristics, such a layer-number dependence of the band gap, chemical doping, large mobilities and the ease of integration with other semiconductors, such as Si or Ge. Transition metal dichalcogenides (TMS) such as MoS_2_ or WS_2_ have bandgaps typically in the visible/UV range, while monolayer graphene is gapless with an interband absorption of ∼2.3% in the visible regime that, due to Pauli blocking in the near-IR to mid-IR, increases in the terahertz because of free carrier intraband absorption [22]. Amongst the many unique properties of graphene, in this review’s framework it is important to highlight a wide carrier concentration modulation (up to ∼10^14^ cm^−2^ [23]) and record mobility at room temperature, e.g., 70000 cm^2^ V^−1^ s^−1^ for industrially relevant chemical vapour deposition [24]. The conductivity *σ* in graphene is normally described by using the Kubo formula, only considering the intraband contribution, e.g. [25, 26];

(1) σintra=i·e2kBTπℏ2(ω+iτ)·[EFkBT+2 ln(exp(−EFkBT)+1)]

where *k*_B_ and ℏ are the Boltzmann and reduced Planck’s constants, respectively; *T* is the temperature, e is the electron charge, *E*_F_ is the Fermi energy and *τ* is the momentum relaxation constant. The momentum relaxation time is normally assumed to be at the order of tens of fs and the temperature at 300 K. Graphene intraband conductivity can be further approximated by neglecting the contribution in the square brackets, thus resulting to a Drude model. Fermi energies of 1 eV or higher can be routinely achieved by chemical ion gating [27], while electrostatic gating reported lower values. In general, graphene supports high concentrated surface plasmons, which can be directly excited and optically modulated in patterned structures [28]. The most prevalent approach is to design architectures where graphene is hybridized with metamaterial resonant features and used as a functional material either via electrical or optical modulation.

### 2.2 Semiconductors

The implementation of semiconductor materials either as bi-dimensional electron gas (2DEG) or as bulk materials was explored as an initial route for modulation [29, 30]. By either tuning electrically or pumping optically above the band gap of the semiconductor, it is possible to achieve an efficient modulation of the free carrier density *N*_s_, e.g., via gate loading or photon absorption, respectively. This active modification of the dielectric constant of the material can again be described via a Drude model with a variable plasma frequency $\omega_{\text{p}}=\sqrt{e^{2}N_{\text{s}}/\varepsilon_{0}\varepsilon_{\text{s}}m^{*}}$ where m* is the semiconductor effective mass, *ε*_0_ and *ε*_s_ are the vacuum permittivity constant and the relative background dielectric permittivity, respectively.

### 2.3 Phase-change materials

These materials undergo a reversable change in resistivity upon the application of several types of stimuli, including thermal heating, electric current excitation, photo excitation, and mechanical strain. Such resistivity change can be either drastic and abrupt (digital) or continuously varying (analogue) and is an ideal platform for THz modulators [31]. In applications where modulation speed is critical (e.g., THz communications), the slow thermo-induced transition is ill-advised, and the preferred option should be photo-induced transition with intense laser pulses whose associated timescale is several picoseconds or less [32], [33], [34], enabling tens of GHz modulation speeds. The most common phase-changing materials for THz modulation is Vanadium Dioxide (VO_2_) [35], which is an alloy family of germanium, antinomy and tellurium together with some dopants, e.g. chalcogenide (e.g., GeTe and GeSbTe) [36] and superconductors [37, 38]. We will expand on these three below. Other phase-changing materials with potential for THz modulation, but are less investigated, include ferroelectrics [39], liquid crystals [40] and liquid metals [41]. The change in resistivity observed in VO_2_ is due to a transition from a monoclinic phase into a rutile phase. At the insulating monoclinic phase, there is a low-density free carrier absorption resulting in high THz transparency. On the contrary, the metallic rutile phase displays a high free carrier concentration (>10^21^ cm^−3^) transforming the VO_2_ into the metallic state thus blocking out THz transmission. Hence, VO_2_ can enable binary switching. The change in material properties of chalcogenides is mediated by nucleation dynamics, enabling a continual varying crystallinity fraction. This in turn, enables an analogue response. Beyond the critical temperature *T*_c_, the resistance of superconductors drops nonlinearly and correspondingly modifies the material’s optical transmission. The high sensitivity of the superconducting state to perturbation arising from incoming electrical and magnetic fields, photons and temperature, make these materials extremely efficient for the design of active terahertz modulators. A complete description of superconductivity is beyond the scope of this review, and it includes a more complex discussion on Cooper pairs, two fluid models to describe the complex conductivity.

### 2.4 Micro-electromechanical-systems (MEMS)

Efficient modulation has been demonstrated with mechanical transformations. Inherently by the underlying physics of metamaterials, reshaping the structural configuration of the metamaterial unit cell or their arrangement can dramatically change their electromagnetic response. This mechanical transformation ability is the natural realm of MEMS, which is a mature technology found in many systems and applications. Within the context of THz metamaterials [42] and modulators [43], [44], [45], this have been limited by fabrication complexity and cost and hence, only modulation speeds at tens of kHz have only been reported so far.

### 2.5 Perovskites

In this fast-evolving research area there are other promising materials, such as perovskites, which have demonstrated remarkable properties for THz modulation. Organic or inorganic halide perovskites have been object of an intensive investigation in the latest years thanks to their remarkable optoelectronic properties, such as long carrier lifetime, high charge carrier mobility and diffusion length, tuneable optical band-gap. One of their greatest advantages consists in their cost-effective processing in solution and compatibility with spin coating processes, whilst stability instead hinders their wide diffusion. Perovskites have been implemented in a variety of optoelectronic devices, from photovoltaics and solar cells to lasers and ultrafast modulators. A full description of their composition, properties and operating principles is beyond the purpose of this manuscript but can be found in reviews [46], [47], [48], [49] or special issue, e.g. [50] and references therein. Their ease of fabrication and compatibility with metamaterials features have been exploited mostly in all-optical modulation schemes, e.g, [51], [52], [53], [54]. Ultrafast optical pulses with energy higher than the bandgap induced free carriers which increase the material conductivity and ultimately damp the metamaterial resonances. The relaxation of the photogenerated carriers normally takes place on hundreds of ps time scale, and it is normally mediated by electron-phonon, other phonon-assisted or trap-assisted processes.

## 3 Terahertz modulators

### 3.1 Amplitude modulators

Amplitude modulation has historically been the first target for dynamic manipulation of terahertz radiation with metamaterial devices see for instance [55]. It is intrinsically more straightforward than frequency modulation, which would require implementing dispersive elements, or polarization modulation, whose designs and fabrication are more complex. Furthermore, amplitude modulators in their wider definition, are the fundamental building blocks for the future THz wireless communication systems (that will likely use OOK (on-off keying) or ASK (amplitude shift keying) modulation schemes according to current roadmaps) or perfect absorbers in coded aperture imaging [56]. A dynamic modulation of the transmitted and/or reflected THz amplitude has been demonstrated in a plethora of configurations; in semiconductors [57, 58], in 2D materials such as graphene [59], [60], [61], [62], [63] or WS_2_ [64] carbon nanotubes [65], thermal changing materials [66] such as VO_2_ [67], [68], [69], [70], MEMS [71], [72], [73], [74], liquid crystals [75] and superconductors [76], [77], [78]. Because of the frequency selective response of most metamaterials (e.g., resonant metamaterials) enhanced amplitude modulators are normally less prone to broadband operation, but in general each approach has its own advantages and disadvantages.

**Figure 2:**
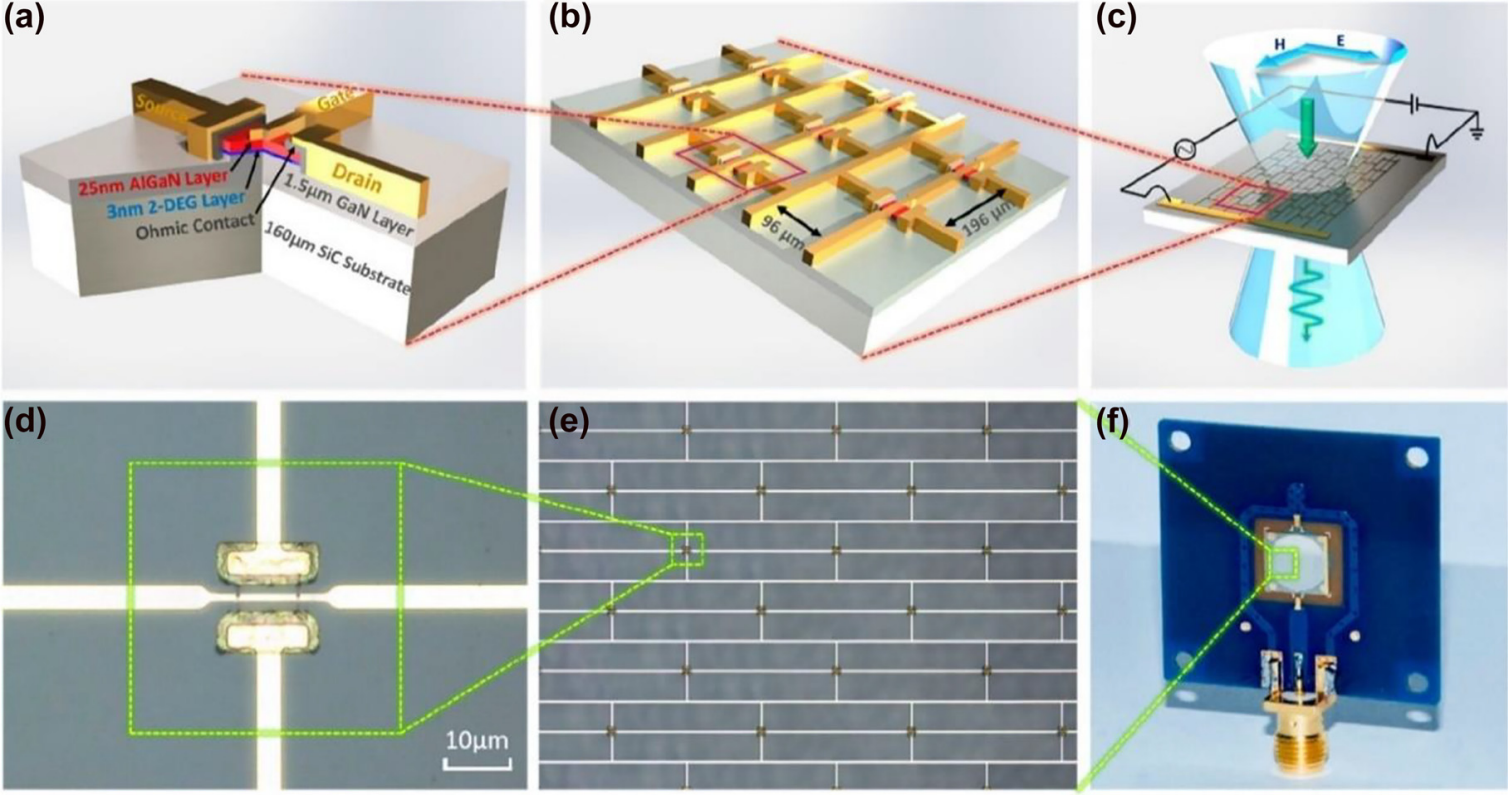
The designed (a)–(c) and fabricated amplitude modulator based on the interplay between SRRs and 2DEG. Reprinted with permission from Y. Zhao et al. Nano Letters 19(11): 7588–7597. Copyright (2019) American Chemical Society, ref. [57].

**Figure 3:**
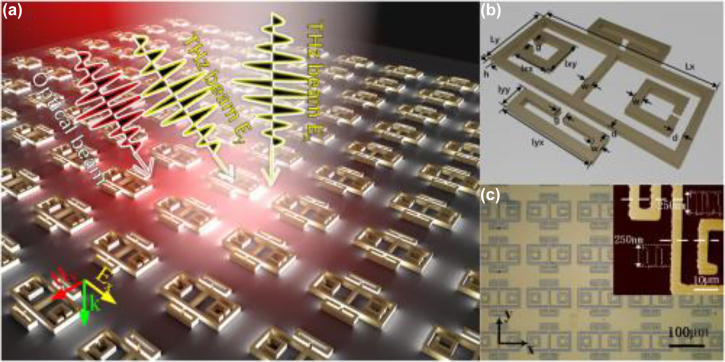
Schematic of the optical pump/terahertz probe arrangement implemented to dynamically tune the resonances supported by the closed and split ring complex metallic metamaterial features onto germanium substrate. Reproduced from Ref [83].

The main FOM for amplitude modulation is the modulation depth (MD), which is defined for the transmission *T* (reflection *R*) as: $\left|T_{\text{on}}\left(R_{\text{on}}\right)-T_{\text{off}}\left(R_{\text{off}}\right)\right|/T_{\text{off}}\left(R_{\text{off}}\right)\times 100\%$ , where the on and off states characterize the extremes of the modulation. The reconfiguration speed of the device is another key parameter that plays a fundamental role in many applications such as wireless communication, or real time imaging. Finally, power consumption or driving voltage, packaging, fabrication technological hurdles are key factors of concern for specific application/experiment. For instance, power consumption will likely be the primary design constraint in future mobile devices. Top-gated SRRs arranged in a staggered netlike array with interacting 2DEG, shown in Figure 2, exhibited a remarkably high modulation depth, up to 93%, due to the shift from individual to collective states [57]. This arrangement can yield superior MDs as the collective structure can be electrically tuned beyond the single metamaterial resonance, while removing the effect of parasitic dipole modes. Furthermore, by carefully acting on the parasitic capacitances and resistances, the device can achieve > GHz modulation speeds, without enhancing the overall device footprint, which affects the free space coupling and the final use of the device in practical applications. Graphene has been implemented as active medium in a myriad of experimental arrangements, such as [11, 25, 59], [60], [61], [62], [63, 79]. The strategy consisting in modulating the interplay between metamaterial units rather than the singular metamaterial resonance was exploited in [59] where tetrameric metallic meta-atoms were separated by ion-gel gated graphene stripes. By varying the graphene conductivity, the adjacent meta-atoms could be electrically connected, thus yielding a wide modulation depth up to ∼50% at 0.61 THz and reporting ∼68° phase modulation at 1 THz as well. Vanadium dioxide has been used in a few experimental configurations as well for achieving dynamic tuning exploiting its wide conductivity phase change at relatively low temperatures (∼67–68 ° C) [67]. Phase changes can be activated in many different ways, from using a straightforward thermal approach to a photo induced transition configuration. In [68] metallic SRRs sitting on top of a vanadium oxide film were used to achieve a dynamic tuning by acting on the temperature of the device yielding ∼35% of modulation depth of the inductor-capacitor (LC) resonance around 0.4 THz when thermally modulated, and ∼15% under a THz pump E-field as high as 3.3 MV/cm. Interestingly, THz pump-induced transition showed a few ps reconfiguration time, thus making it compatible with ultrafast modulation. It should, however, be noted that this speed is indicative of the ultimate physical limit attainable by the material rather than a direct measure of the device’s reconfiguration capability. A continuous wave laser emitting at 808 nm was successfully implemented for the dynamic tuning of hybrid metamaterial/VO_2_ metasurface, yielding 80% modulation depth with 2.5 W impinging IR power [69]. The modulation was recorded at different frequencies using an acousto-optic modulator and showed a clear degradation at around 1 MHz. In [80, 81], the phase-transition was driven electrically, which is a major step toward THz system-on-chip. The metamaterial’s unit cell consisted of three horizontal metal lines and a vertical metal bar on the Si_3_N_4_–VO_2_–Si_3_N_4_–Si composite substrate [80] and of an omega-shaped Aluminium particle with two inner concentric split-rings made of VO_2_ and Aluminium on sapphire [81]. Maximum modulation depths at ∼0.8 THz reached 99 and 71% for a 300 mA bias current difference, respectively. Other phase-change materials that are gaining momentum for analogue amplitude modulation are chalcogenides, namely Ge_2_Sb_2_Te_5_, abbreviated as GST [36]. In [36], asymmetric metallic split ring resonators (ASSR) array fabricated onto a GST film supported a Fano-like resonance at ∼0.7 THz which was actively damped by a combination of thermal and electrical means resulting into a 100% modulation depth (defined there with respect to the Fano resonance strength, peak-to-peak). Unfortunately, modulation speed was in the timescales of seconds or higher. To address the slow modulation speed, optical excitation was used in the same reference; complete recovery of the Fano resonance was observed at after ∼19 ps with a pump fluence of 636.6 μJ/cm^2^, which means a modulation speed in the range of few tens of GHz; the modulation speed was subsequently quantified in [82] to be ∼33 GHz. Germanium has attracted a great attention for the realisation of THz modulators lately, mostly because of a faster recombination carrier lifetime (on the order of ∼ps) compared to Si, thus compatible with ultrafast operation. In [83], a MEMS metamaterial metallic array, comprising closed and split rings was fabricated on top of a Ge layer, as shown in Figure 3. The overall THz response of the array, which exhibit polarization anisotropy, aimed to engineer a transparency transmission window between two coupled resonances, particularly useful in light of active group velocity dispersion. The carriers induced by ∼800 nm fs-pulses shunted the capacitive gaps hence affecting the interplay between the different resonators and the final optical response. For pulse energy of 1 mJ/cm^2^, a ∼40% and a ∼45% modulation depths were recorded at 0.79 THz and 0.9 THz for *E*_*x*_ and *E*_*y*_ incoming light polarization with an overall recovery time of ∼3 ps. A similar approach has been used also in Ref. [58] where the electromagnetic induced transparency (EIT) window was calculated by using an inverse design algorithm in order to obtain the desired optical features. An optical pump power of 2.2 mJ/cm^2^ yielded about 50% modulation depth at 0.67 whilst the whole EIT figure could be modulated in approximately 15 ps. ASSR arrays fabricated onto a Ge film were providing a different dispersive Fano-like optical response which could be actively damped in a similar way, of about 20% MD around 0.9 THz under 1.2 W optical pump power with 17 ps recovery time [84].

Perfect absorbers play a significant role as well in imaging and detection [85]. The standard engineering of perfect absorbers is based on a Salisbury screen arrangement wherein a resistive layer is placed approximately at *λ*/4 from a ground plane (i.e., perfect electric conductor). By replacing the resistive layer with a metasurface that enables acting on the effective permittivity and permeability of the arrangement, the distance between the top layer and ground plane can be reduced significantly, while the surface impedance of the arrangement is matched to the vacuum impedance *Z*_0_ = 377 Ω, yielding perfect absorption [86]. The implementation of a suitable tuneable medium enables a dynamic modulation. The introduction of a metasurface, basically a grating, into a graphene-based absorber [25] managed to reduce the requirements on the Fermi energy tuning thanks to the E-field enhancement in the graphene-loaded slits. In fact, perfect absorption could be achieved at approximately 0.3 eV, rather than >1 eV more demanding Fermi energies, and a modulation depth of 25% at 0.43 THz was demonstrated. Huygen’s metasurface absorbers [87] based on Si disk resonator arrays arranged on top of a low refractive index matrix have been demonstrated as an interesting and relatively simple arrangement for the dynamic tuning of transmission/absorption. The tuning mechanism was based on optical inducing charges with a near infrared pulsed beam, in a standard optical-pump-terahertz-probe experiment. The absorption around 1 THz was modulated from 97.5 to 40% with a remarkably low pump fluence of 140 μJ/cm^2^ where higher fluence have minor effect on the amplitude modulation. Vanadium dioxide based metasurfaces are constantly suggested for perfect absorbers or other kind of modulation schemes [86, 88, 89], also in combination with graphene.

MEMS are known for their large MDs in the THz range. They are normally based on upstanding metamaterial elements with respect to the surface of the substrate forming a relative angle, which can be modified by applying a suitable voltage to release the fabrication stress between the different components. Broadband operation and size of the single elements are matter of concern, which limits their use for some applications, such a coded-apertures for single pixel imaging where sizeable pixel are required. In Ref. [72], the authors reported a MEMS array formed of multipixel (singularly addressed) metallic (Cr–Cu–Cr) cantilever arrays partly anchored onto a dielectric parylene C substrate whose inclination angle can be dynamically tuned by applying a suitable voltage. The authors achieved >50% average modulation depth remarkably over a range spanning from 0.97 THz to 2.28 THz and operating with 37 V. Integrated elements capable of reproducing logic gates such as AND and OR together with coding metamaterials are hot topic in the THz field, and metamaterials offers the efficiency, miniaturization and integration scalability to address these challenges as for instance in [66]. In Ref. [73], Xu et al. fabricated a dual band polarization sensitive metamaterial MEMS array with a perfect absorber metamaterial structure sensitive to the two resonances selected by the MEMS cantilever, thus realising proof-of principle logic gates OR and AND at 0.33 THz and 0.88 THz, respectively. The integration capability of this complex design opens new perspective in terms of applications, e.g., of programmable devices for future wireless communications beyond the amplitude modulation results. The winding shape cantilever array (WCM) is based on deformed electrothermal actuators whose vertical displacement can be modified by applying a voltage as high as 20 V. A DC voltage bias variation from 11 to 20 V modified the 0.33 THz resonance intensity by 23% whilst the dynamic tuning range of the 0.88 THz one was 45%.

Superconductor metamaterials have historically been studied [90, 91] as they offer resistive low losses below the critical temperature *T*_c_, thus higher *Q* factors, they exhibit intrinsic dispersive features because of the dynamic inductance, and can be tuned in several different ways [92], [93], [94]. In fact, superconductivity can be destroyed electrically, magnetically, with strong optical pump below and above the superconducting energy gap *E*_g_, on top of the straightforward thermal mechanism. Photon energies below *E*_g_ are normally required in order not to destroy the Cooper pairs, thus setting THz and sub-THz as the natural spectral region for investigating these materials. Nb and NbN are the most common superconductors, but their low *T*_c_, about 14–15 K for NbN, limits their frequency range below 1 THz. Cuprates superconductors, such as YBa_2_Cu_3_O_7−*x*_ (YBCO) and Bi_2_Sr_2_CaCu_2_O_8+*δ*_ (BSCCO), exhibits *T*_c_ (*T*_c_ ∼85 K for BSCCO) beyond the boiling point of liquid Nitrogen (77 K) and therefore present more relaxed experimental conditions and tuning ranges. Thermal tuning of BSCCO SRRs fabricated onto a sapphire substrate is reported in Figure 4 from [95], yielding a 75% MD around 0.5 THz. YBCO (*T*_c_ = 90 K) SRRs thermal tuning of the transmission spectra was demonstrated in literature, e.g., in [94] where an MD of 55% around 0.6 THz was reported. NbN dispersive EIT features were also dynamically tuned by using powerful THz pulses, as intense as 30 kV/cm, yielding similar MD of ∼50% at 0.5 THz [93]. Optical pumping with photon energies higher than the Cooper binding energy resulted in the ultrafast (∼5 ps) destruction of superconductivity in YBCO fabricated ASRRs on sapphire [96], and therefore on a drastic reduction of their Q-factor. The (partly) restoring of the Fano and dipole resonances supported by the ASRRs takes place on time scale of tenths of ps, depending on the impinging fluence, thus achieving a complete ultrafast modulation cycle. The maximal MD reported was 86% at a fluence of 955 μJ/cm^2^. All optical approaches are normally used also for perovskite-based metamaterial THz modulators. Photoinduced conductivity in perovskite materials would effectively damp the resonances supported by metamaterial metallic features. Amongst the remarkable optoelectronic properties mentioned in the introduction, halide perovskites have been investigated for the realisation of active THz devices thanks to their high level of compatibility with standard fabrication techniques, granting also the realisation of flexible structures, and the low fluence required to achieve high modulation depths. A few experiments reported after ultrafast rise up of the photoconductivity, relaxation times of the free carriers varying from ∼100 ps to >ns. A 2D perovskite/metamaterial device showing 20 ps free carrier relaxation time has been recently demonstrated thanks to exciton confinement in self assembled quantum well structures [97] reporting also a modulation depth > 90% around 0.7 THz for a fluence of 0.25 mJ/cm^2^. CMOS technology integrated with metasurfaces has also been reported with remarkable modulation performances such as 25 dB modulation depth and 5 GHz reconfiguration speed, on top of digitally reconfigurable capabilities operating at 300 GHz [98]. CMOS approach is anticipated to play a key role in the lower part of the sub-terahertz spectrum but is not easily foreseeable to scale to higher frequencies.

**Figure 4:**
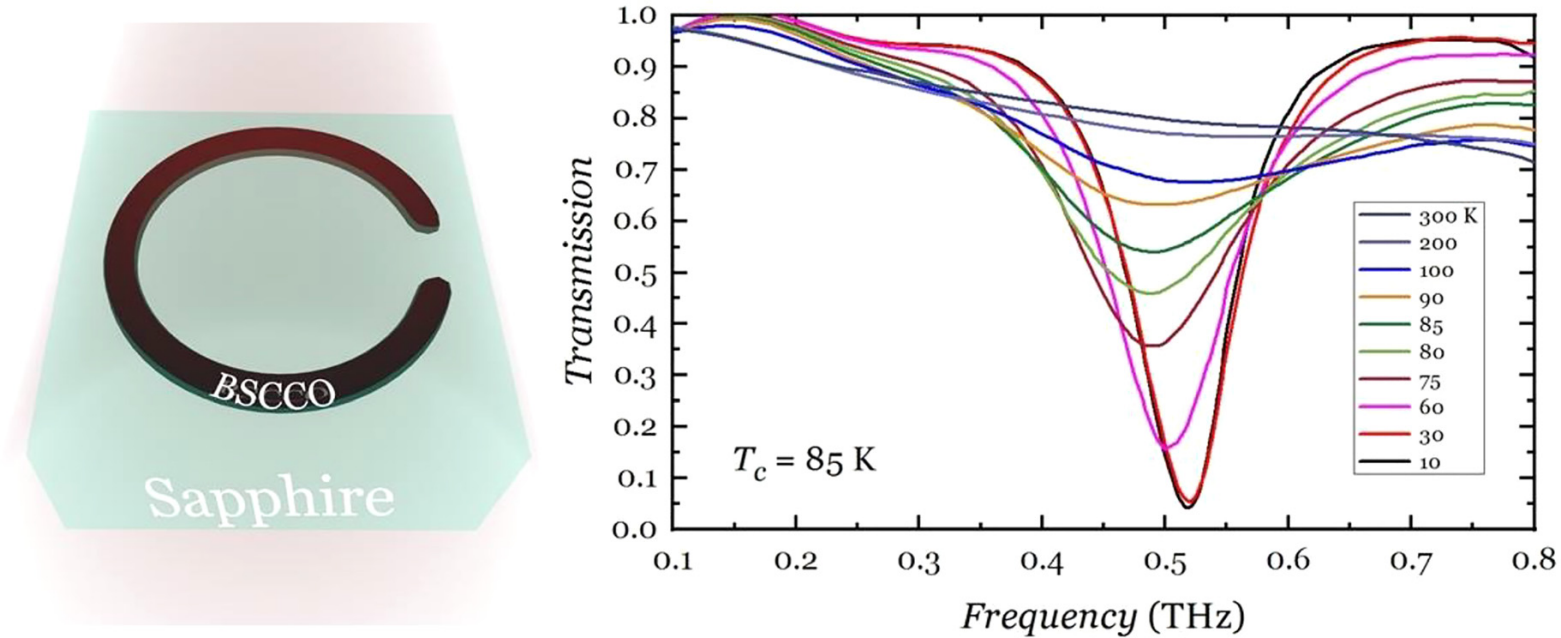
Thermal tuning of terahertz resonances supported by BSCCO SRRs, from Ref [95].

A summary of the main approaches pursued to achieve amplitude modulation is reported in Table 1, together with the main FOMs.

**Table 1:** THz amplitude modulators designs and FOMs.

Design	Method	MD	Frequency (THz)	Speed	Reference
Gated SRRs	Semiconductor	93%	0.34	3 GHz	[57] Zhao et al. (2019)
	Electronic				
Molecular meta-atoms	Graphene	49.3%	0.61	//	[59] Jung et al. (2018)
	Electronic				
Hybrid SRRs	VO_2_, thermal,	35%	0.4	//	[68] Liu et al. (2012)
	fs THz pump	15%	0.4	>100 GHz	
Hybrid SRRs	VO_2_, IR pump	80%	0.34	MHz	[69] Zhang et al. (2014)
Cut-wire metamaterials	VO_2_, thermal,	65%	0.6	//	[70] Wen et al. (2010)
Cut-wire metamaterials	VO_2_, electrical	99%	0.8	//	[80] Hu et al. (2021)
Hybrid omega-shaped metamaterials	VO_2_, electrical	71%	0.79	//	[81] Jiang et al. (2021)
ASRRs on GST	Ge_2_Sb_2_Te_5_, thermal, electrical	100%	∼0.7	Hz, ∼33 GHz	[36] Pitchappa et al. (2019) and [82] (2021)
Metamaterials on Ge	Semiconductor	∼40% (*x*)	0.79	∼300 GHz	[83] Sun et al. (2020)
	Optical pumping	∼45% (*y*)	0.9		
Metamaterials on Ge	Semiconductor	50%	0.67	66 GHz	[58] He et al. (2021)
	Optical pumping				
ASRRs on Ge	Semiconductor	25%	0.9	∼58 GHz	[84] Lim et al. (2018)
	Optical pumping				
Perfect absorber	Graphene, electrical	25%	0.43	//	[25] Chen et al. (2020)
Perfect absorber	Silicon, optical	47%	1	//	[87] Fan et al. (2018)
Cantilever array	MEMS	∼50%	∼0.97–2.28	//	[72] Kappa et al. (2019)
	Electrical				
WCM array	MEMS	23%	0.33	//	[73] Xu et al. (2021)
	Electrical	45%	0.88		
Superconductor SRRs	BSCCO SC, thermal	75%	0.5	//	[95] Kahlor et al. (2017)
Superconductor SRRs	YBCO SC	55%	0.6	//	[91] Chen et al. (2010)
	Thermal				
Superconductor EIT	NbN	∼50%	0.5	//	[93] Zhang et al. (2017)
	THz pulses				
Superconductor ASRRs	YBCO SC, fs optical pulse	∼44%	∼0.4	∼25 GHz	[96] Srivastava et al. (2018)
ASRRs on perovskite	2D perovskite, fs optical pulses	>90%	∼0.4	∼50 GHz	[97] Kumar et al. (2020)
CMOS metasurfaces	Electrical	25 dB	0.3	∼5 GHz	[98] Venkatesh et al. (2020)

### 3.2 Frequency modulators

Research into terahertz frequency modulators is driven by the few applications where an active control on the metamaterial resonance dispersion is required. These include but are not limited to: spectroscopic applications, frequency division multiplexing imaging [99] and communications fundamental research into strong light–matter interaction [100], and ultrafast quantum electronics where an active engineering of the group delay dispersion is required. By far the most used technique to investigate dispersive THz modulators is based on broadband time domain spectroscopy (THz-TDS), commonly based on fs pulse lasers impinging onto an Auston switch or nonlinear crystals for emission and detection.

**Figure 5:**
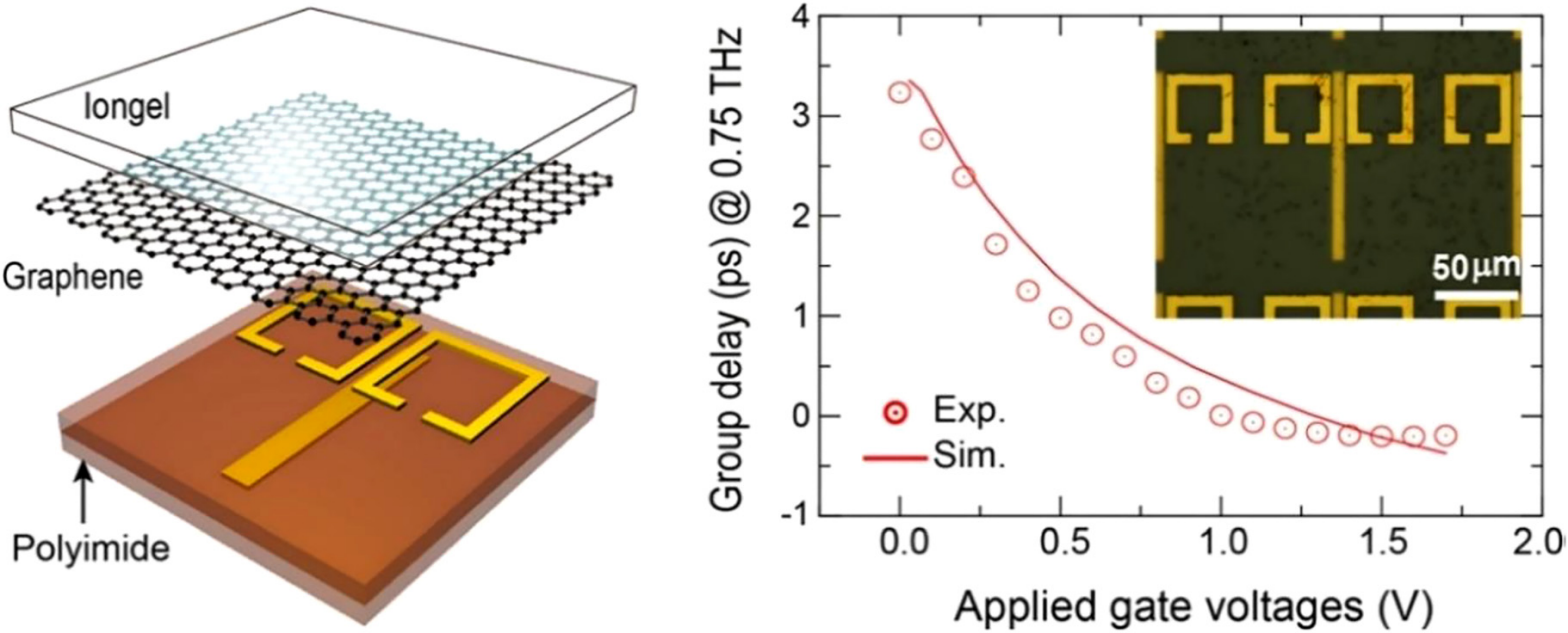
EIT hybrid metamaterial graphene arrangement for the dynamic tuning of the group delay. Reprinted with permission from TT Kim et al. ACS Photon. 5(5): 1800–1807. Copyright (2018) American Chemical Society, ref. [102].

**Figure 6:**
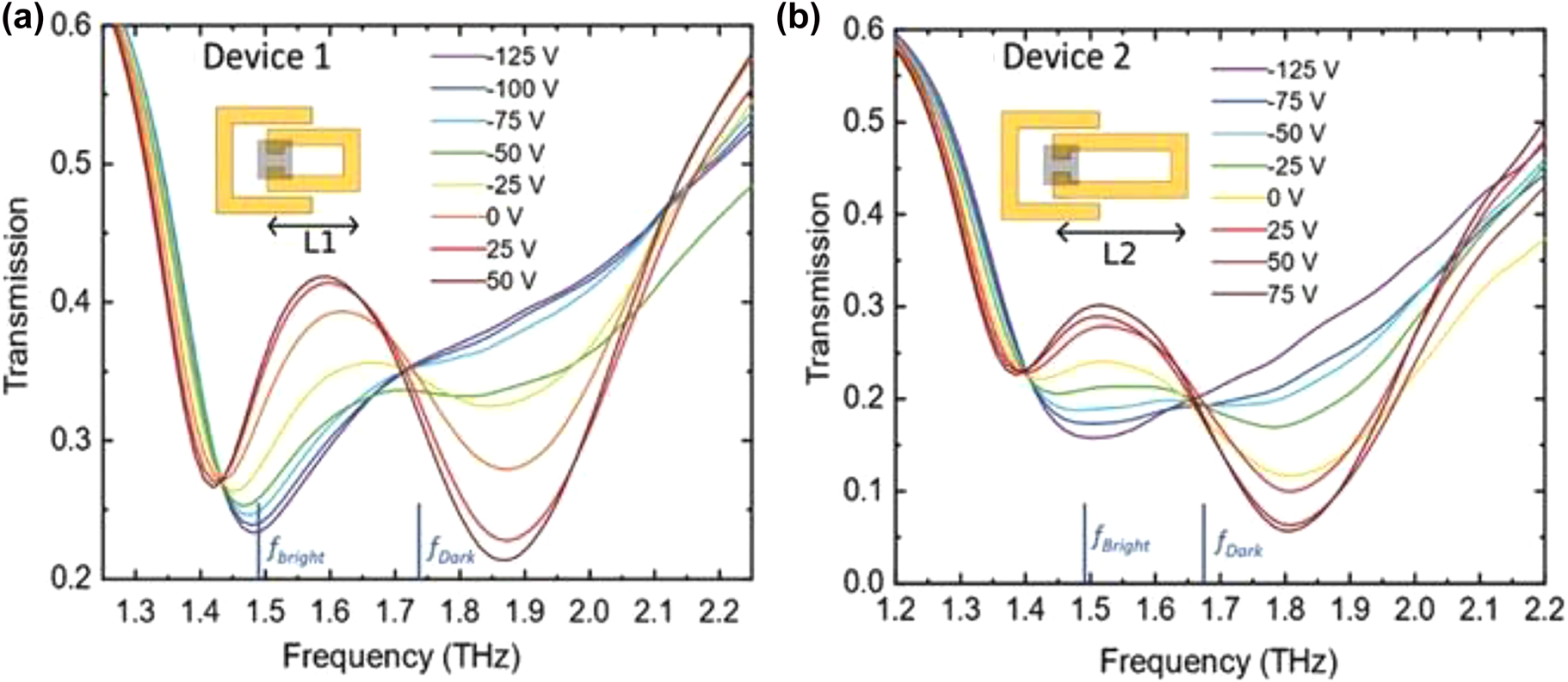
EIT metamaterial array using graphene as functional material. The interplay between bright and darker resonators induces two strongly correlated dips in transmission. By electrostatic gating graphene patched which are shunting the dark resonators, the overall frequency and amplitude response of the device are modulated, from Ref. [104].

Superconductor metamaterials are intrinsically dispersive thanks to their kinetic inductance and together with the MEMS, a direct change of the resonant frequency conditions can be made, e.g., via capacitance tuning. Photoinduced change in Si island capacitances in SRR arrays also proved to be an efficient method in the pioneering article from Chen et al. [101] and achieved a frequency change of about 20% around 1 THz, under 500 mW pump power at 800 nm wavelength. A different approach that has gained popularity in recent years is based on the EIT equivalent of the quantum phenomenon originally investigated in atomic physics. The EIT equivalent metamaterial optical response can be reproduced by coupled optical or electronic oscillators, leading to the build-up of a transparency window between the two coupled resonances. By combining this phenomenon with an extreme modification of the dispersion properties, a slow light effect results due to an alteration of the group velocity of the propagating pulse. This is important because active control of the group delay and slow light has great implication in fundamental research, due to the enhanced light–matter interaction and nonlinearity, and for practical applications, e.g., for the active dispersion compensation in mode-locked lasers. The realisation of the EIT metamaterials dynamic tuning is normally achieved by damping either the Q-factor of one resonance or the mutual interaction between the single elements. The two main FOMs as reported in Table 2 are the relative frequency shift ( $\Delta f/f_{\text{max}}\times 100\%$ ) of the resonance and the group delay *t*_g_ in ps. The group delay is defined as: $t_{\text{g}}=-\text{d}\varphi /\text{d}\omega$ where the d*ϕ* is the relative transmission phase with respect to a reference, e.g., air, and *ω* is the angular frequency. Hence metamaterials whose resonant frequency changes under an adequate stimulus are naturally addressing group delay modulation as well. Figure 5 shows an EIT metamaterial arrangement that consists of a bright resonator (the cut-wire) separated by a polyimide dielectric spacer from two dark ring resonators, which cannot be directly excited by the incident polarized E-field, but rather by the coupling with the first metallic element [102]. An ion-gel gated graphene layer allowed the control of the SRRs’ Q-factor. The EIT design was elegantly described by two coupled harmonic oscillators’ model where the EIT metamaterial arrangement did not show a significant frequency shift, but rather optimized to achieve slow light and tunable group delay with low voltage, 3.3 ps with <2 V respectively. A similar approach was implemented in [103], having bright and dark resonators fabricated on the same substrate and with graphene patches shunting only the capacitances of the SRRs. Electrostatic gating yielded a 100 GHz continuous frequency tuning at ∼1.8 THz. Figure 6 shows another EIT arrangement, which uses two SRRs coupled via mutual capacitance [104] where the Q factor of the dark resonator could again be actively tuned by altering graphene conductivity. This resulted in a 120 GHz continuous frequency tuning at ∼1.4 THz, and a group delay modulation of –0.2 ps.

**Figure 7:**
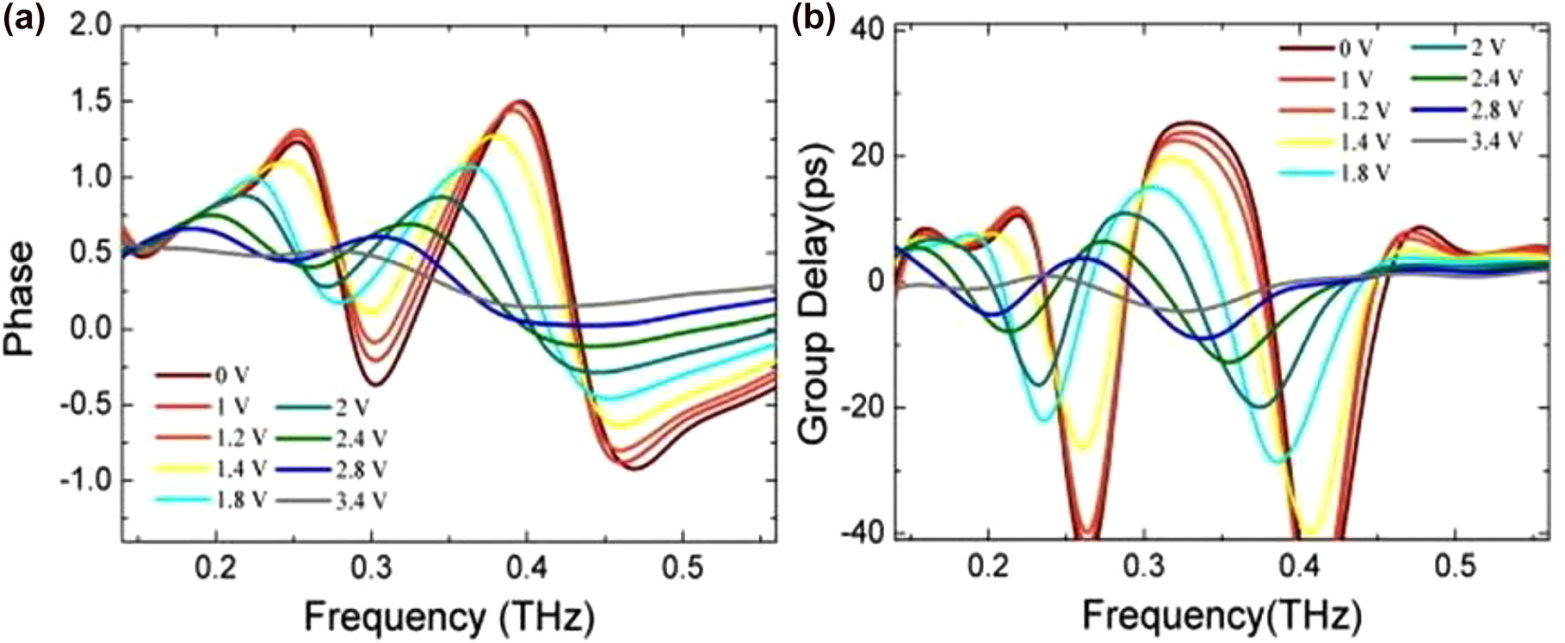
Phase and group delay modulation for the electrically tunable NbN EIT array at 4.5 K. Reprinted from C. Li et al. Appl. Phys. Lett. 2021; 119: 052602, with the permission of AIP Publishing, Ref. [105].

Superconductor metamaterials show intrinsically dispersive features due to the dynamic inductance and were successfully demonstrated with a few different materials, e.g., high-*T*_c_ cuprates such as TBCCO [77], YBCO [91], BSCOO [95], and NbN [105] or also combining superconductor/metallic metamaterial features [106]. Thermal frequency tuning for SRRs fabricated in BSCCO and YBCO were reported to be 7 and 35% at 0.52 THz and 0.48 THz maximal frequency, respectively. In Ref. [77] the authors reported an EIT-like design fabricated in TBCCO which exhibits on top of the LC and dipole (DP) also a Josephson plasmon resonance (JPR), thus opening up new opportunities for the design of Josephson-based emitters, receivers and nonlinear ultrafast devices. The first two resonances could be separately excited by selecting orthogonal incident incoming polarized E-field, whilst the JPR resonance had similar trend for both polarizations; the corresponding frequency shifts are summarised in Table 2, together with FOMs and designs for the latest active dispersive metamaterial designs. Figure 7 shows the active group delay modulation achieved by Li et al. by electrically tuning NbN concentric SRRs EIT arrays. The superconductivity in the outer rings was electrically modulated by inducing Ohmic losses thus modifying the overall array’s optical response. It is worth noting that the frequency shift reported, 32%@0.34 THz, and the record group delay modulation of 25.4 ps at the transparency window (i.e., 0.34 THz) have been achieved only with a few volts biasing, thus paving the way for real-time applications. However, more advantageous higher *T*_c_ SCs might require higher voltage to drive these devices. Significant ultrafast (a few ps) group delay modulations have been reported also in metallic metamaterials on Ge substrate by optical pumping [58, 83] showing larger group delay modulations: >−17 ps around 0.65 THz and and >−6 ps around 0.67 THz under a pump fluence of 1 mJ/cm^2^ and 2.2 mJ/cm^2^, respectively. MEMS continue to be investigated as well for the realization of frequency dispersive tunable devices [107, 108]. In [107] a tunable bandpass filter based on reconfigurable cantilever integrating complementary split ring resonator (CSRR) is reported. The T-shaped cantilever arrays were fabricated with Al_2_O_3_/Al over the Si substrate. The residual stress, which can be released by applying a suitable voltage, curves the cantilever, thus changing the resonance. Under 24 V voltage bias the resonance changes from 0.64 THz to 0.37 THz, thus achieving a 42% frequency shift. The method is very effective in achieving a wide frequency tuning on a CMOS compatible platform, even though frequency tuning appears to be more compatible with a bistable, rather than a continuous modulation. Table 2 reports the latest results on THz dispersive modulators.

**Table 2:** Dispersive THz modulators methodologies and relative FOMs.

Design	Method	Frequency shift (%)	*t*_g_ (ps)	Reference
EIT	Graphene	//	3.3@0.75 THz	[102] Kim et al. (2018)
	Electronic			
EIT	Graphene	6%@1.8 THz	//	[103] Kindness et al. (2019)
	Electronic			
EIT	Graphene	8%@1.4 THz	−0.2@1.9 THz	[104] Kindness et al. (2018)
	Electronic			
SC SRR	BSCCO	7%@0.52 THz	//	[95] Kalhor et al. (2017)
	Thermal			
SC SRR	YBCO	35%@0.48 THz	//	[91] Chen et al. (2010)
	Thermal			
SC SRR	TBCCO, thermal	17%@0.42 THz (LC)	//	[77] Guo et al. (2021)
		11%@0.98 THz (DP)		
		31%@0.65 THz (JPR)		
SC SRR	NbN, electrical	32%@0.34 THz	24.5@0.34 THz	[105] Li et al. (2021)
SRRs	Metamaterial on Ge optical	//	>−17@0.65 THz	[83] Sun et al. (2020)
Inverse metasurface	Metamaterial on Ge optical	//	>−6@0.67 THz	[58] He et al. (2021)
CSRRs	MEMS, CMOS	42%@0.64 THz	//	[107] Pitchappa et al. (2021)
Plasmon induced transparency	SRRs/CRRs on perovskite	//	−1.5 ps@1 THz	[53] Zhou et al. (2019)

### 3.3 Polarization modulators

Dynamic tuning and manipulation of terahertz light polarization states is intrinsically elusive, and underpins a range of applications. These include polarization sensitive imaging and spectroscopy, multichannel communication and sensing. Polarization modulation comprises optical activity (OA) or circular dichroism (CD), describing the rotation of the polarization plane and the ellipticity conversion when the light is passing through a medium, respectively. The left- and right-circular polarization states, LCP and RCP respectively, interact differently in chiral media, but natural chirality is normally poor and requires thick samples for long wavelengths. Because of a lack of active materials in the terahertz range, different solutions need to be adopted in order to achieve efficient polarization modulation. Amongst the various strategies, the metamaterial approach is by far the most adopted as meta-atoms offer the versatility, efficiency and capability to engineer an artificial EM response needed for polarization devices and for realising artificial chirality. A more general theoretical description of chirality can be found in [14], [15], [16], [17] and here we aim to recall the main concepts and parameters needed for quantifying dynamic tuning. On top of arbitrary mirror and rotational symmetry, metamaterial can be combined into multi-layers, yet retaining high efficiency and integration capability. A linear incident polarization can be described as the superposition of RCP and LCP, having the same amplitude and phase. When entering a polarization sensitive medium, the complex refractive index seen by these two components *n* ^+^(*n* ^−^) for RCP (LCP) is different and therefore the corresponding complex transmitted components *T* ^+^(*T* ^−^) will change as well. A difference in the real part of the refractive index results in a rotation of the optical polarization plane (OA). A difference in the imaginary parts between *n* ^+^ and *n* ^−^, so a different absorption, instead modifies the polarization from linear to elliptical (CD). These effects are normally quantified starting from the complex transmission states for the two light spin states by the polarization angle *θ* and the normalized ellipticity *ε*, respectively, where:

(2) $$\theta =\frac{1}{2}\cdot \left[\text{Arg}\left(T^{+}\right)-\text{Arg}\left(T^{-}\right)\right]$$

and:

(3) $$\varepsilon =\frac{\left|T^{+}\right|-\left|T^{-}\right|}{\left|T^{+}\right|+\left|T^{-}\right|}$$

The *T* ^±^ components describing in circular coordinates can be retrieved form the cartesian components of the incoming E-field, *E*_i_^*x*,*y*^ and the transmitted ones *E*_t_^*x*,*y*^ by using Jones matrix formalism, e.g., as in [98].

(4) $$\left(\begin{array}{c} E_{\text{t}}^{x}\\ E_{\text{t}}^{y} \end{array}\right)=\left(\begin{array}{cc} T_{xx} & T_{xy}\\ T_{yx} & T_{yy} \end{array}\right)\left(\begin{array}{c} E_{\text{i}}^{x}\\ E_{\text{i}}^{y} \end{array}\right)=T\left(\begin{array}{c} E_{\text{i}}^{x}\\ E_{\text{i}}^{y} \end{array}\right)$$

Where the elements having the same subscripts are meant to describe the co-polarized transmission components and the ones with different subscripts the cross-polarized contribution. The transmission matrix **T** in cartesian components can be transformed in circular base in **T**_**circ**_ where:

(5) $$T_{\mathrm{cir}}=J^{-1}TJ\;\text{where}\;J=\frac{1}{\sqrt{2}}\left(\begin{array}{cc} 1 & 1\\ i & -i \end{array}\right)$$

The definition of the ellipticity provided in (2) has the immediate advantage to be correlated to the ration of the two semiaxes in the polarization ellipse, varying between 0 corresponding to linear polarization to ±1 corresponding instead to pure circular polarization states. It is possible to find alternative definitions to quantify CD, the most popular one is by using *η* instead:

(6) $$\eta =\frac{1}{2}\sin^{-1}\left(\frac{{\left|T^{+}\right|}^{2}-{\left|T^{-}\right|}^{2}}{{\left|T^{+}\right|}^{2}+{\left|T^{-}\right|}^{2}}\right)$$

The *η* value spans from ±45° for perfect circular polarization to 0° for linear polarization. Ideally, it would be desirable to address OA or CD independently, but effectively these two effects are difficult to be separated. As a general rule, if OA is to be targeted, the design should focus onto the maximization of off-diagonal elements in the transmission matrix. Conversely, the optimization of CD, e.g., linear to circular conversion, aims to have a 90° phase difference between the two orthogonal components, hence common experimental arrangements are frequently based on multilayer metasurfaces.

**Figure 8:**
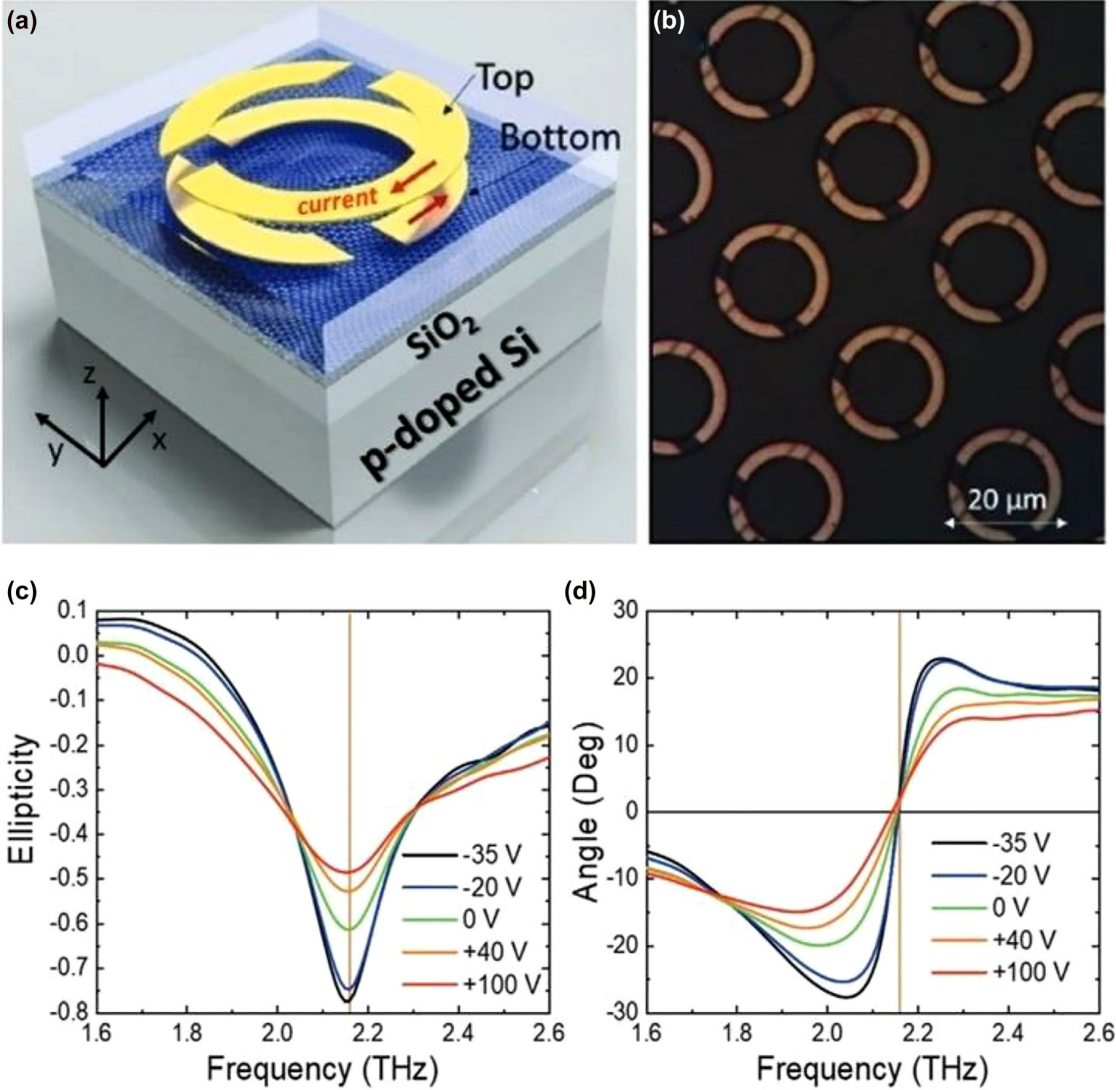
Double layer chiral modulator based on SRR metasurfaces rotated by 90°. (b) top view optical image of the final device. (c) Ellipticity and (d) rotation angle of the incoming polarization as a function of the applied voltage. Adapted and reproduced from Ref. [110].

**Figure 9:**
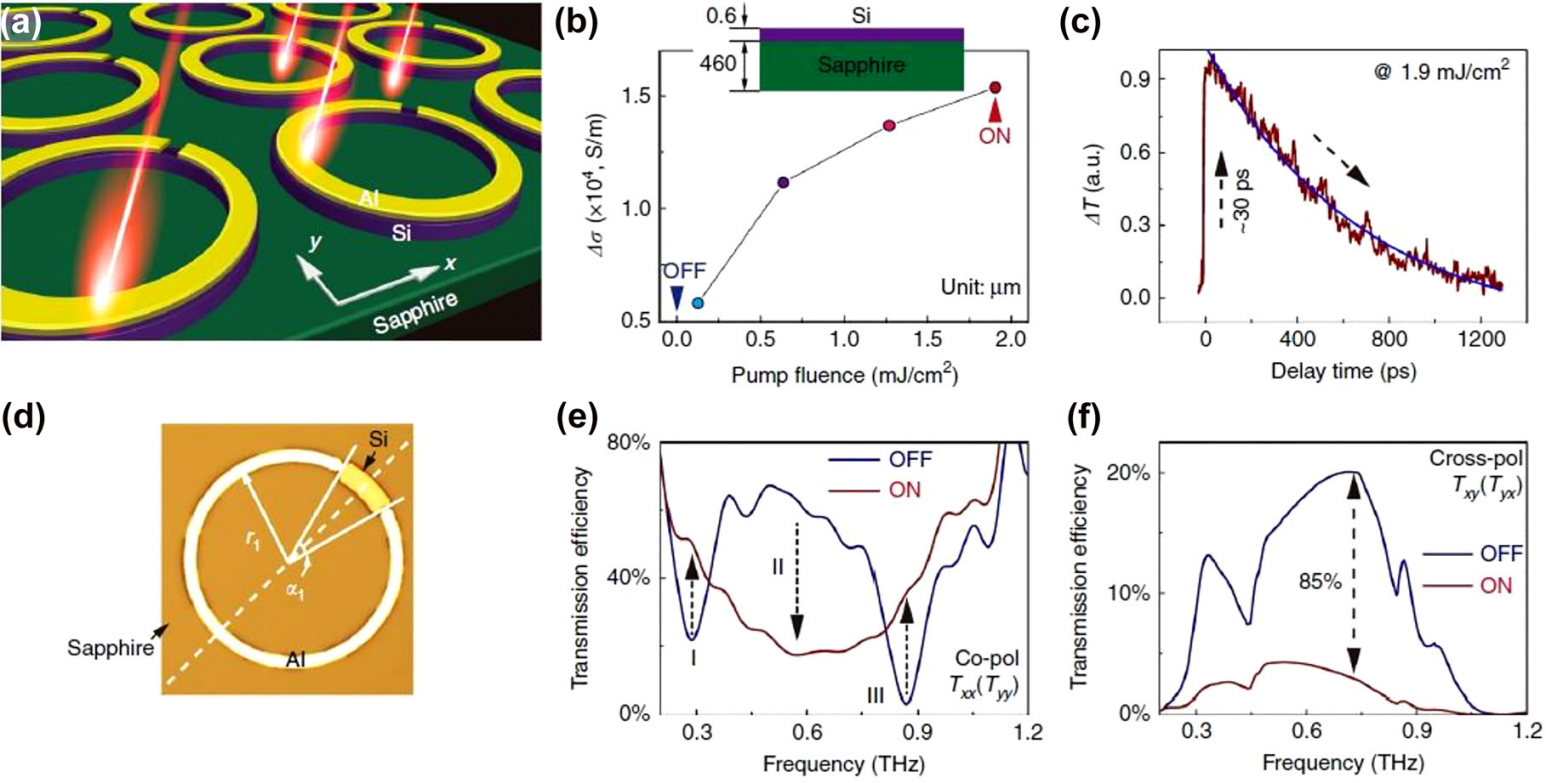
SRR array fabricated in silicon/metal onto a sapphire platform. These structures supported different optical modes upon incident THz radiation polarized at 45° with respect to the symmetry axis of the SRR. These resonances can be actively damped by an opportune near infrared sub-ps pulse around 800 nm, strongly absorbed by the Si in the gap. Adapted and reproduced from Ref [112]

**Figure 10:**
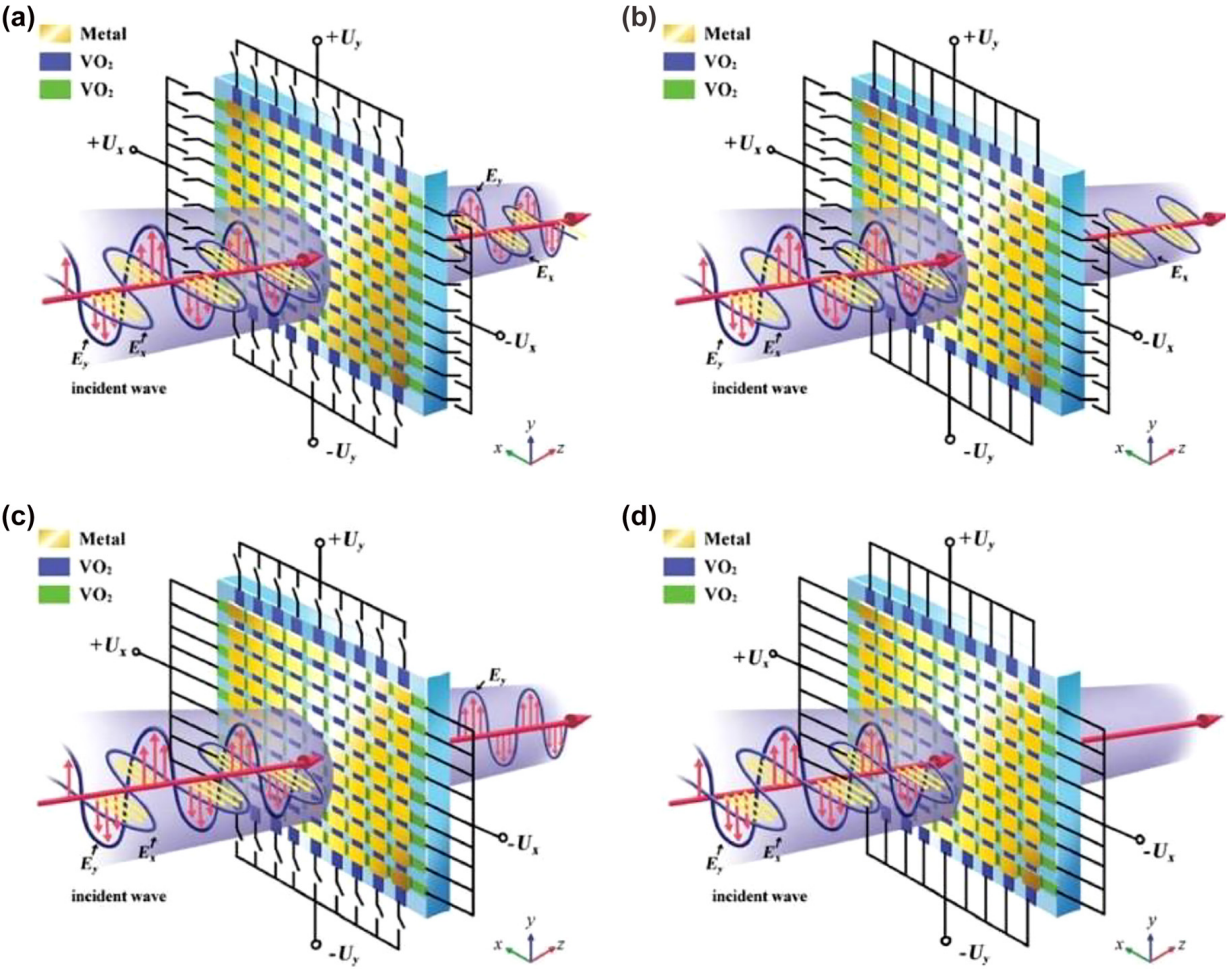
Tuneable hybrid VO_2_/metadevice enabling polarization selection. In the high conductive state, the device reflects the E field component along the direction of the applied voltage. Reproduced from Ref. [114].

**Figure 11:**
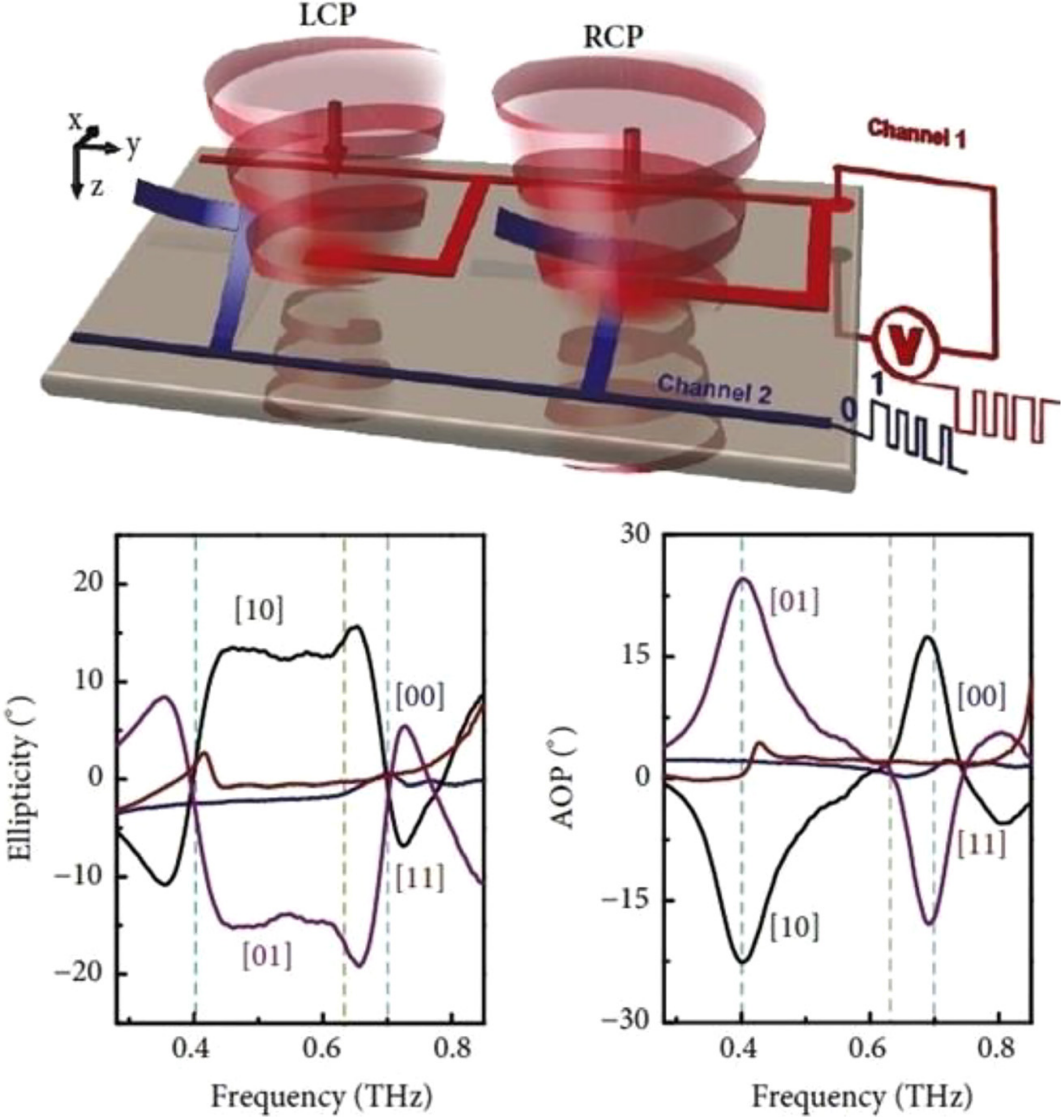
Digital reconfigurable MEMS metasurface based on doublefold L-shape cantilevers.The independently voltage driven cantilever arrays will provide symmetric modulation of the ellipticity and of the OA, here defined as twice as the θ defined in Eq. (1). Adapted and reproduced from Ref. [121]

The main approaches to achieve a modulation, consists in either a mechanical change of the metamaterial structure, using MEMS based structures, or in the introduction of an active medium interacting with the metamaterial resonances, such as 2DEG, graphene, photosensitive materials, e.g., Si, thermal phase changing materials, such as VO_2_, or liquid crystals. In Ref [109], Kim et al. used a classical arrangement aiming to exploit mainly CD based on two different conjugated double Z metamaterial surfaces separated by a polyimide dielectric. The top surface is in contact with a graphene monolayer, whose conductivity can be modulated via ion-gel with a few Volts gate bias. By electrically varying the conductivity, an active damping was achieved for RCP, whilst minimally affecting the LCP. The dynamic Δ*ε* factor quantifying the CD is approximately 0.82 at the resonance of 1.1 THz, whilst an OA of ∼10°, with negligible CD was recorded far from resonance at 1.4 THz. A double layer design using two metasurfaces rotated by 90°, separated by a polyimide dielectric layer and using graphene as an active medium is showed in Figure 8 [110]. The frequency was centered at ∼2 THz, thus allowing integration with QCLs. Single metallic metasurfaces whose unit cell is composed of bright and dark resonators, reminiscent of the EIT, were also implemented targeting purely active OA achieving a ∼20° at 1.75 THz [103]. The tunability was obtained by acting on the interplay between dark and bright resonators and specifically by modifying the dark resonators’ Q factor via graphene conductivity gating. Electrostatic gating of graphene allows access to a reduced conductivity range compared to ion-gel but can cater for larger reconfiguration speeds, e.g., >5 MHz. Another theoretical strategy [26] relying on graphene based metasurfaces is to exploit multiple reflections to boost broad polarization conversion. This modulation scheme, though, is based on the variation of the incident angle, makes this approach less appealing for the realisation of active devices. Optical pump has been proposed as viable theoretical solution for the modification of multi-layered graphene metasurfaces, under 980 nm continuous wave pumping [111]. Numerical simulations report a η modulation of 20° with 10 mW/mm^2^ pump power at 0.76 THz. Optical pumping of Si embedded in metamaterials has also been widely demonstrated in the literature as an efficient method for achieving large polarization modulation depths. In Ref. [112], Cong et al. used arrays of metallic SRRs shunted by silicon gap to achieve an active polarization modulation upon optical pumping with near–IR sub-ps pulse source with a pump fluence of ∼2 mJ/cm^2^. By positioning the SRR array with the symmetry axis rotated with respect to the incoming linear incident E-field, the polarization sensitive modes supported by the metamaterials were actively damped, whilst the other ones were not affected by the change in conductivity. The co- and cross-polarization transmissions are shown in Figure 9, together with the measured reconfiguration speed measurement, this latter one dominated by the long carrier recombination time in Si (typically ∼ 1 ns). The overall ellipticity measurement yielded a modulation of *η* ∼ 30° at 0.84 THz. It should be highlighted that these authors also managed in a different set of experiments to engineer the dispersion of the transmitted wavefront by changing the phase yielded by adjacent SRR units, such as to spatially separate the output for co- and cross-polarization, *de facto* realizing an efficient polarizing beam splitter. Liquid crystals, usually in their nematic phase, are largely used as functional material in the whole EM spectrum due to their large optical anisotropy (typical Δ*n* ∼ 0.2–0.3) that can be flexibly manipulated by thermal, optical, electric, and magnetic fields with large MDs. The combination of liquid crystal with metamaterials aims to provide a further boost to their dynamic range and to their efficiency. In [102] the authors merged an asymmetric metasurface composed on multiple concentric SRRs with a liquid crystal cell sandwiched between two silica layers. Electrical contacts at the sides of the liquid crystal cell provided the voltage necessary to change the material birefringence. The overall chiral response reported wide CDs and OAs for the co-transmitted and cross-transmitted polarizations, *ε*_co,cross_ and *θ*_co,cross_ as reported in detail in Table 3, which summarises the main results for polarization modulators. For instance, for the co-transmission the CD could be varied between −0.3 and 0.8 at 0.76 THz and ∼30° at 0.83 THz. A fast reconfiguration speed of this device is limited by the viscous resistance of liquid crystals and their bulkiness. Practical applications should also consider that the achievable dynamic range and the voltage required, which can go as high as 300 V. Recently, increasing attention was placed to the implementation of phase-change materials, such as VO_2_, which exhibits a transition from insulator to metal at around ∼67° with an increase in conductivity > 10^4^ S/m. The transition can be enabled either thermally, electrically or via optical pumping. Hybrid VO_2_/metamaterials have been proposed for CD and OA [114], [115], [116], [117], [118], [119] holding promise of high efficiency. In Ref. [114], the authors proposed the integration of metallic patches with VO_2_ stripes, which can be independently electrically biased, as shown in Figure 10. This arrangement operates efficiently in ∼0.33–0.4 THz and allow selective rejection of one polarization depending on the direction of the voltage applied with a 25 dB extinction ratio between the two polarizations. Effectively, it operates as a voltage tuneable grid polarizer. Figure 10 shows its implementation as polarization rotator with *θ* = 45° but in principle it can work up to <90° to the expenses of a sharp increase in power losses. It should be mentioned that on top of the intrinsic speed limitations connected to a thermal tuning mechanism, this device also requires an activation voltage of 130 V. Exploiting Babinet’s principle [120] for the design of a hybrid metamaterial and VO_2_ configuration on sapphire substrate, narrowband reconfigurable quarter-wave plates were realised by thermal (300 and 370 K) [117] and electrical stimuli (0 and 180 mA bias current) [118]. The ability to switch between RCP and LCP output was successfully demonstrated at ∼0.6 THz in both works with no information on the modulation speed provided. Such helicity inversion was recently reported on cross-shaped patches connected with VO_2_ bridges on sapphire substrate without the need to resort to Babinet’s principle, which enabled enhancement of the bandwidth of operation to 0.25 fractional bandwidth [119]; due to the thermal activation, the modulation speed was in the timescale of minutes. MEMS are well suitable for the engineering of reconfigurable chiral response. In [121] Cong et al. utilises two conjugated L-shape bimorph cantilever MEMS metasurface. The L-shaped unit cell is formed of metallic Al over a dielectric Al_2_O_3_ dielectric substrate and due to residual stress tends to bend and to self-assemble into a 3D anchored helix. The stress can be removed by applying a reasonably low voltage (10 V) thus restoring the planarity. This is an intrinsically chiral device, and interestingly bistability offered by the independent voltage control of the differently oriented L-shape micro-cantilevers not only provides a symmetric way to select RCP or LCP light, but can also be exploited for coding information. However, this elegant approach requires a complex fabrication and has a limited reconfiguration speed and a relatively limited modulation depth. A symmetric Δ*η* of 30° at around 0.65 THz and Δ*θ* > 22° at 0.4 THz, as shown in Figure 11 were reported. Semiconductors chiral metamaterials are also used as well. Recently, in [122] Fan et al. realised a complex architecture presenting magneto optical spin mode conversion in InSb crystals. The interplay between the asymmetric metal metasurface and the anisotropy induced by a longitudinal B-field allowed a few configurations for the conversion of linear polarization into RCP and LCP and the direct conversion between circular polarization states. This approach exhibits in some configurations, remarkable polarization conversion efficiencies, such as the almost 100% conversion from RCP to LCP around 0.7 THz, but the tuning mechanism based on magnetic field excitation strongly limits practical use. A non-exhaustive list of the recent progresses made for polarization modulation, together with the main FOMs is summarised in Table 3.

**Table 3:** Recent developments in polarization modulation and relative FOMs.

Design	Method	OA or CD range	Frequency (THz)	Speed	Reference
Conjugated Z metamaterials	Graphene	Δ*θ* = 10°	1.4 THz	//	[109] Kim et al. (2017)
	Electronic	Δε ∼ 0.82	1.1 THz		
Conjugated metamaterials	Graphene	Δ*θ* = 10°	1.9 THz	//	[110] Kindness et al. (2020)
	Electronic	Δε ∼0.45	2.1 THz		
EIT metamaterials	Graphene	Δ*θ* = 20°	1.75 THz	>MHz	[103] Kindness et al. (2019)
	Electronic				
Conjugated graphene multilayers	Graphene	Δ*η* = 20°	0.76	//	[111] Masyukov et al. (2020)
	Optical				
SRRs on silicon	Silicon, optical	Δ*η* ∼ 30°	0.84	>1 GHz	[112] Cong et al. (2018)
Asymmetric metamaterials		Δ*ε*_co_ ∼ 1.1	0.76	//	[113] Ji et al. (2021)
	Liquid crystals	Δ*ε*_cross_ ∼ 1.4	0.73		
	Electrical	Δ*θ*_co_ ∼ 30°	0.83		
		Δ*θ*_cross_ ∼ >60°	0.83		
Grid polarizers	VO_2_, electrical	Δ*θ* = 90°	∼0.33–0.4	//	[114] Wong et al. (2020)
Babinet metamaterials	VO_2_, thermal	Δ*θ* = ±90°	∼0.6	//	[117] Nakata et al. (2019)
Babinet metamaterials	VO_2_, electrical	Δ*θ* = ±90°	∼0.66	//	[118] Nakanishi et al. (2020)
Cross patches with VO_2_ bridges	VO_2_, thermal	Δ*θ* = ±90°	0.9	Minutes	[119] Kobachi et al. (2021)
L-shape cantilevers	MEMS	Δ*θ* ≥ 22°	0.4	//	[121] Cong et al. (2019)
	Electrical	Δη ∼ 30°	0.65		

## 4 Selected applications

A full review of all the applications in the terahertz range is beyond the purposes of this review and it is a matter which has been extensively discussed in several books [1], [2], [3], [4], [5], [6], [7], [8], [9]. At the same time there are a few selected research and industrial sectors, which greatly benefit from the recent flourishing of efficient terahertz modulators: namely terahertz wireless communications, sensing and quantum electronics.

### 4.1 Terahertz wireless communications

Terahertz wireless communication is universally recognized as the future platform for high data rate (100 Gbit/s – 1 Tbit/s) transmissions underpinning 6G and beyond. There has been a clear trend in the recent years driven by the increasing demand for hungry data rate applications, such as augmented reality, medical imaging, rack-to-rack communications, e.g., in supercomputers, military, automotive and satellite communications, kiosk downloading, indoor links and so on and so forth [123], [124], [125]. The constant need for an unallocated frequency range inevitably pushes toward the carrier frequencies into the THz regime, following Shannon’s theorem. Furthermore, a higher frequency range compared to standard wireless communications is more favourable in terms of antenna efficiency and compactness. Next generation THz wireless communications strives to be fast, efficient optoelectronics enabled and programmable coded devices operating at high carrier frequencies. The metamaterial approach seems naturally to address all these requirements. It is well-known that by increasing the carrier frequency from the RF range the transmission will be affected, particularly beyond 1 THz, by atmospheric attenuations. Depending on the different weather conditions, these losses might reach 10–100 dB/km, thus limiting *de facto* the range for the THz spectral range, to indoor, meter distances or even nanoscale. However, the near field range has a few peculiar applications not limited only to device-to-device communications, but also communications between different objects, e.g., sensors integrated on the same chip, such as transistors, memories where data extraction is complex. Wireless communications in the photonics range would offer a wider bandwidth but would suffer from even higher attenuation levels especially in atmospheric conditions, e.g., foggy and heavy rain, compared to THz communications, and higher demands in terms of beam directionality. The THz attenuation defines the frequency range of wireless communications, e.g., mid-field rather than near-field communication, and the applications targeted consequently as well. So far, D and G bands up to 300 GHz wireless emission have been successfully covered by the progress of electronic sources and technology. Most notably, it is worth mentioning CMOS technology achieving at 300 GHz > 100 Gb/s [126]. However, these results have been achieved using CMOS with channels on the order of tenths of nm and using mixer stages. It does not seem foreseeable a further channel frequency increase for this technology significantly beyond 500 GHz. Resonant tunnelling diodes as well as uni-travelling carrier photodiodes are now emitting at room temperature beyond 1 THz, with power levels typically of the order of 1–10 uW around 1 THz, steeply declining at higher frequencies. Their intrinsic direct modulation makes them suitable for wireless communications and can also be externally modulated.

**Figure 12:**
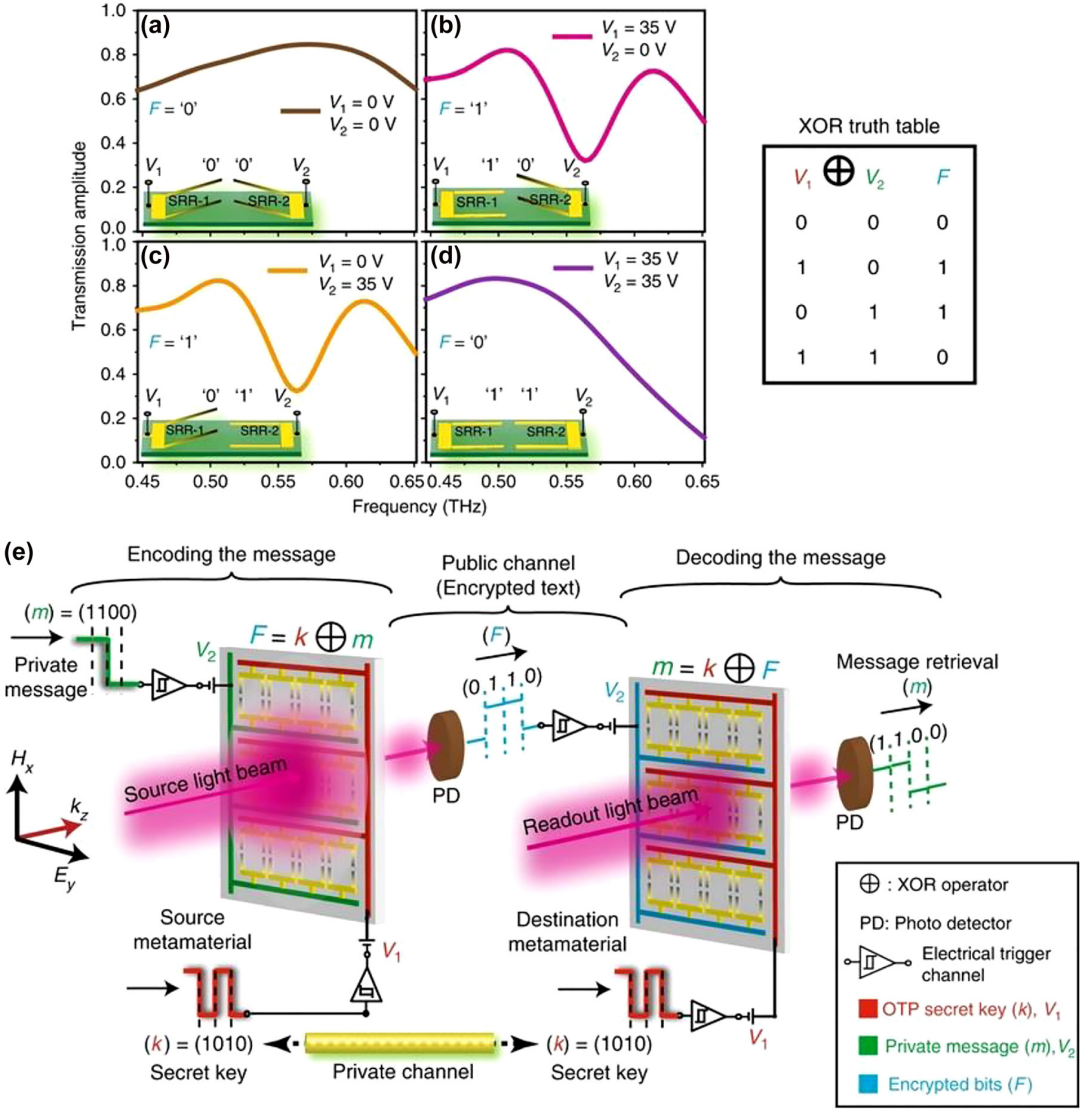
MEMS Fano metasurfaces for (XOR) logic operation and their potential use in cryptographic wireless communication networks, reproduced from Ref. [129].

**Figure 13:**
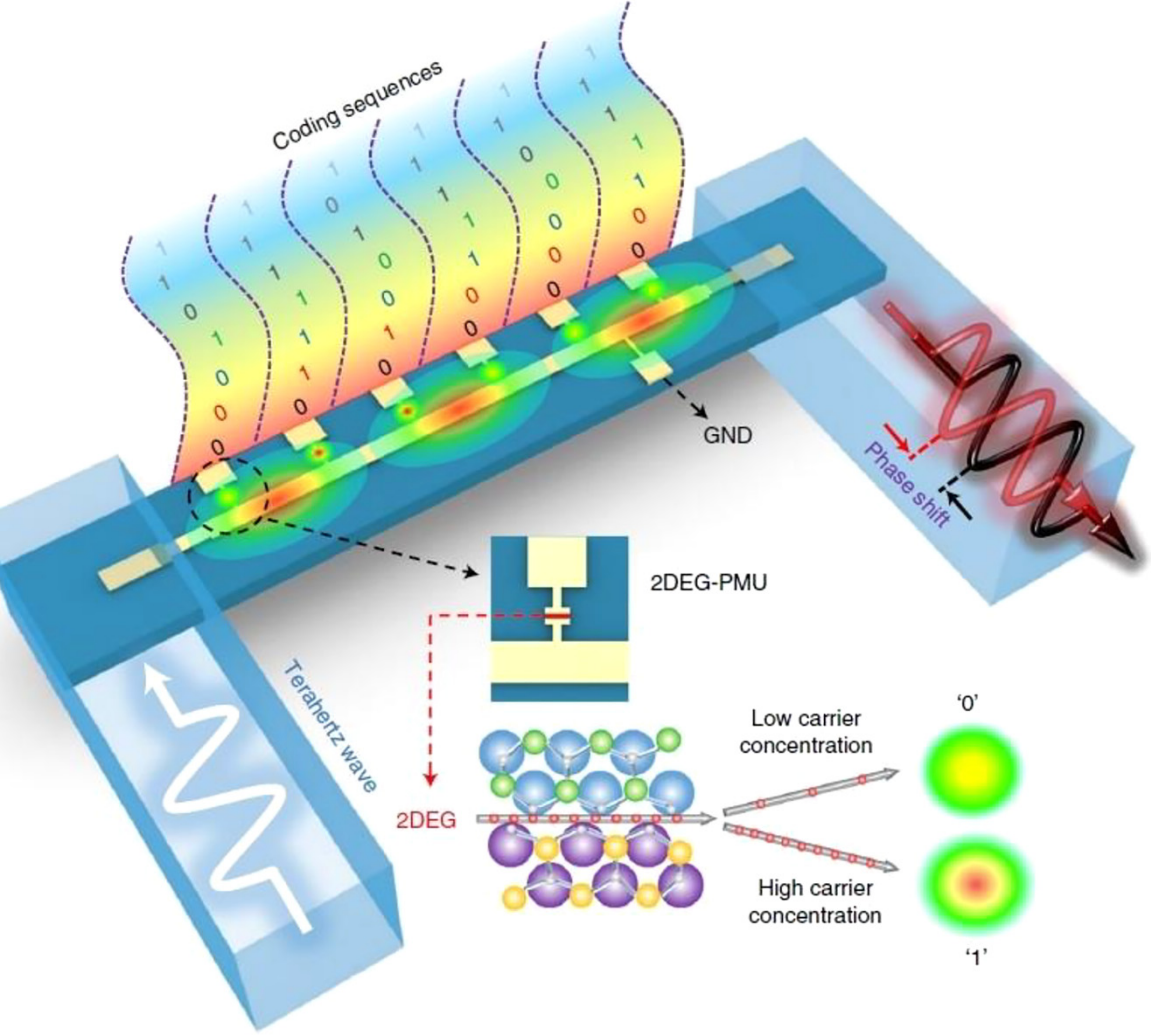
Phase coding at THz frequencies based on integrated metamaterials and 2DEG, Reprinted by permission from Springer Nature Customer Service Centre GmbH: Springer Nature, Nature Photonics, H. Zeng et al. Nat. Photon. 15: 751–757. Copyright (2021), ref. [130].

The key for unlocking the broad unallocated bandwidth and high carrier frequencies beyond 300–500 GHz necessarily passes through the developing of an adequate integrated optoelectronics circuitry capable of supporting a fast response, compatible with the whole THz spectral range and possessing high efficiency [127]. The metamaterial approach seems the more promising route for addressing all these demands and for realising external fast optical modulators [128] and integrated logic devices [129, 130]. Metamaterial naturally satisfies the requirement for high efficiency, due to their strong light concentration and miniaturization capability, thus allowing lower power consumption. The spectral versatility and wide configuration range granted by this approach are other key factors, which make metamaterials particularly suitable in the design of a THz wireless platform. A major breakthrough for wireless communications has been achieved with the introduction of programmable, digitally coded metamaterials [131, 132]. This approach has been first proposed in the RF spectrum, but it can directly be extended to the THz range [133]. It is based on the exploitation of the reconfigurable properties of metamaterial arrays where every single unit cell or pixel can be independently addressed electronically. Beam steering devices, spatial light modulators, phase modulators and field programmable metasurfaces as gate arrays have been demonstrated using this concept. Figure 12 shows a reprogrammable THz modulator based on two independently electronically driven MEMS SRRs [129]. The MEMS-based metallic metasurfaces exhibit independent Fano resonance that can be used in a multiple input arrangement to enable logical gates in the far field such as XOR, XNOR, and NAND. The proof-of-principle implementation of secure OTP key for message encryption highlights again the versatility of these functional devices. The coding metamaterial approach has been recently used also for encoding phase digital bits into a single chip [130] in the lower part of the terahertz spectrum. The authors combined a microstrip transmission line with a multichannel digital capacitive metamaterial coupled to a 2DEG and arranged in series, as showed in Figure 13. The different logic states “0” and “1” corresponds to low and high carrier concentration levels, whilst the cascade arrangement increase the dynamic range up to 55° at 0.27 THz with insertion losses of 5–6 dB and low average phase error (2°–5°). The particular arrangement allows to achieve a robust phase control with minimal amplitude modulations, whilst the low RC constant suggesting high reconfiguration speeds.

### 4.2 Terahertz metamaterial sensing

**Figure 14:**
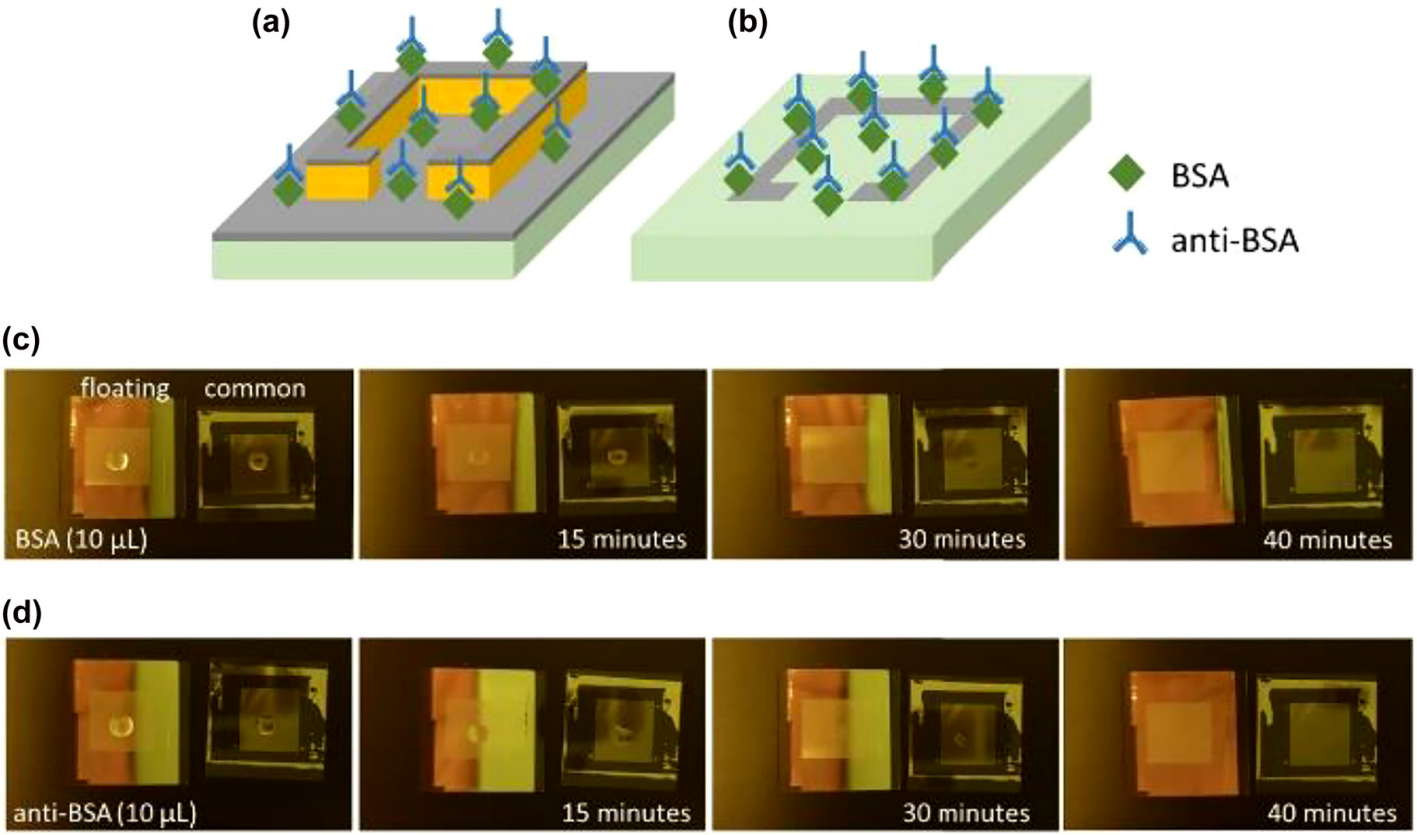
Protein binding of BSA and anti-BSA on floating THz mea, from Ref [145]

**Figure 15:**
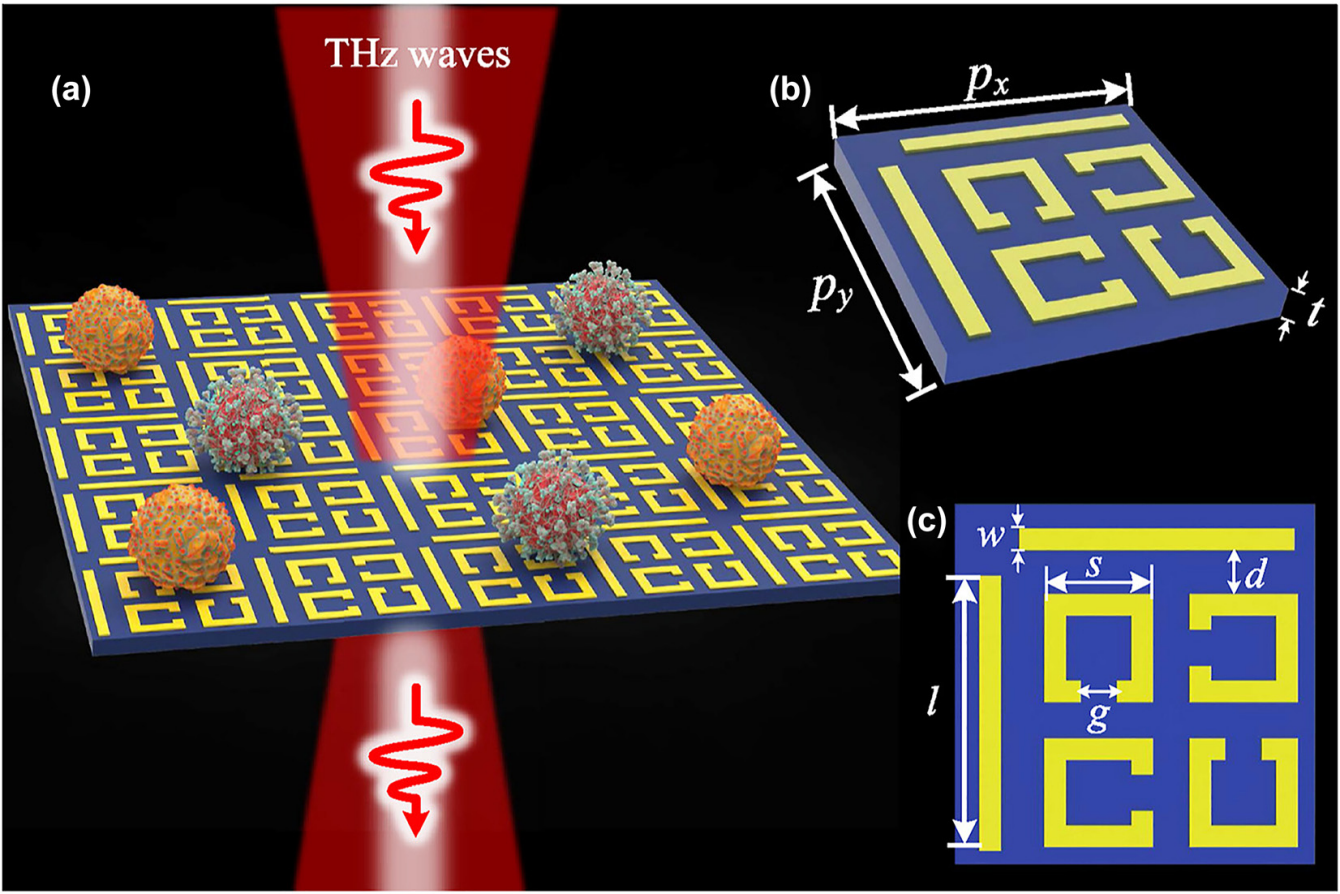
Illustration of a metamaterial biosensor where cells are cultured on and probed by an incident THz beam. Reprinted from Biosens. Bioelectron. 185: 113241. J. Zhang et al. Highly sensitive detection of malignant glioma cells using metamaterial-inspired THz biosensor based on electromagnetically induced transparency. Copyright (2021), with permission from Elsevier, ref. [146]

Significant development and progress have been made on applying terahertz metamaterials for real-life sensing application. Reviews on this subject matter have been covered extensively [19, 134]. This is an area where rapid progress has been made. As a testament of interest and developments in this area, in 2021 alone there are already at least 50 publications on the subject matter! Here we will highlight some key developments made since the latest review made. In electromagnetics, the reflection and transmission at the interface between two homogeneous media are determined by the continuity of field components. When this interface is modified by the addition of periodic sub-wavelength resonators by means of micro-fabrication, this results in the encoding of target material properties in the resulting reflection and transmission signals. The signal change in turn translates to measurable changes in resonant peaks designed at discrete frequencies and the Q factor of these resonances, which is taken as the ratio of the resonant frequency over the full width at the half maximum of the resonances. Other benchmarks include detection sensitivity and FOMs commonly described as resonant frequency with change in refractive index and the product of sensitivity and Q factor, respectively. It should be noted that detection sensitivity can also be expressed in concentrations such as mol/L or g/mol. From a technological point of view, there has been developments made employing single or multi-resonances for localised sensing. In particular, a perfect absorber made of all-dielectric metasurface with periodic sub-wavelength InSb micro-rod array is proposed for simultaneous temperature and refractive index sensing. Temperature dependence is achieved from using InSb with electrical properties being dependent on the surrounding thermal radiation [135]. In Ref. [136] Yao et al. exploited two resonances, Fano and EIT for sensing thin polyimide films with thicknesses down to 180 μm and different liquids on polymer-based flexible substrate, suitable for commercial adoptions [19]. Exploiting impedance matching, a three-band narrowband perfect absorber is proposed that consists of a sandwich structure of a hollow Dirac semi-metallic top layer, followed by a photonic crystal slab and a gold layer beneath [137]. Quad-band perfect absorbers have also been proposed where the bands can be adjusted by an external stimulus to modulate the electrical conductivity of the photosensitive Si [138]. Similarly, a toroidal split ring resonator flanked by two asymmetric C resonators is used to achieve multi-band transparency with a Q factor ∼3 numerically [139]. The potential use of toroidal dipoles is also investigated using cantilever structures, which can be tuned by thermal actuation of the bending angle in the out-of-plane direction with a toroidal dipole intensity increasing by five orders of magnitude [140]. The prospect of further enhancing the Q factor of the toroidal resonance to an extreme value to 10^5^ is investigated in [141] by distorting the symmetry in the bound state in the continuum. For most of these new designs, only modelling results are presented. There has also been progresses made in applying THz metamaterials for practical sensing applications particularly in the field of biomedicine [142]. Specifically, [143] presented a molecule-specific aptamer hydrogel-functionalized biosensor to detect trace thrombin-induced variations. The optimized biosensor demonstrated specificity for serum sample assays and a detection limit down to 0.40 pM in the human serum matrix. A high electric-field enhanced absorber based on bow-tie triangle ring metal microstructure array is proposed to detect the breast cancer marker carbohydrate antigen 125 [144]. To enhance refractive index sensitivities for proteins, a method of floating resonators by patterned photoresist is proposed [145]. As shown in Figure 14, this method achieves a higher sensitivity due to the fact that near-field is no longer distributed over the substrate surface. The provided sensing demonstrations on bovine serum albumin (BSA) and on the protein binding of BSA resulted with a low concentration, smaller than 0.150 μmol/L. To include resonance magnitude changes as part of detection, variations in the EIT resonance frequency and magnitude were used to resolve mutant and wild-type glioma cells cultured on the metamaterial at any cells concentrations without introducing antibody (see Figure 15) [146]. Multi-resonances metamaterials have also been applied for the label-free identification of early-stage cervical cancerous tissues where resonant frequencies shift are used for detection [147]. Compared to single resonance detection, doubling the number of resonances results in higher identification accuracy. On the theme of label-free detection, is the probing of the hydration dynamics of Aβ aggregates, used as therapeutic and diagnostic biomarkers, in liquids where the use of a metamaterial further enhances the detection sensitivity down to 1 nM [148]. Beyond measuring properties of the resonance such as frequency shift and magnitude, an interesting approach to exploit chirality is demonstrated for amino acids aqueous solution. In particular, the chiral enantiomers of low concentrations could be distinguished by observing differences in THz polarisation parameters at characteristic frequencies [149]. The most recent results and relevant FOMs for sensing with THz metamaterials are presented in Table 4.

**Table 4:** recent sensing FOMs and approaches with THz metamaterials.

Sensing mechanism	Benchmarks (sensitivity THz/RIU, quality factor, figure of merit 1/RIU)	Application	Additional information	Source
Two resonance (Fano and EIT) absorption sensing	60 and 100 GHz/RIU	Liquids	Flexible substrate	[136] Yao et al. (2022)
		Polyimide film thicknesses < 180 μm		
4 band at discrete frequencies perfect absorber			External stimulus can adjust 2–4 bands	[138] Wang et al. (2022)
High Q factor multi-resonances by a toroidal resonators and two ‘C’ shaped resonators	Q factor ∼ 3		Modelling only	[139] Bhattacharya et al. (2021)
Dual-resonance and triple-resonance, polarisation sensitive	Average sensitivity, Q factor, and FOM of TTM device are 0.186 THz/RIU, 231 and 187		Dual/triple-resonances can be switched by changing the distance in the resonators. Modelling only	[150] Wen et al. (2021)
Multi-pole resonances			Tunable toroidal dipoles by thermal actuation	[140] Chen et al. (2021)
Single resonance by spoof surface plasmons	Detection limit of 0.40 pM	Trace analysis of human α-thrombin	Aptamer hydrogel-functionalized THz metamaterial.	[143] Zhou et al. (2021)
Bow-time triangle metal microstructure array THz absorber	498 GHz/RIU	Breast cancer marker carbohydrate antigen	Two kinds of biosensors presented	[144] Cui et al. (2021)
	(20 μg/mL)			
Floating metamaterial to ensure near field not distributed over the substrate	532 GHz/RIU	Sensing protein binding of bovine serum albumin	Patterned photoresist to float SRRs at height of 30 um from substrate	[145] Silalahi et al. (2021)
	0.15 μmol/L			
Three-band narrowband perfect absorber based on a hollow Dirac semi-metallic layer above, a gold layer below and a photonic crystal slab in the middle	Q factor of 106		Tunable range is 0.06 THz by tuning Fermi energy from 60 to 140 meV	[137] Li et al. (2021)
	0.1525 THz RIU^−1^		Modelling	
	FOM 4.26			
All dielectric InSb metasurface as THz perfect absorber	Q factor 53.24	Temperature and RI sensing	Modelling	[135] Cheng et al. (2021)
	Temperature dependent sensing 1043.3 GHz/RIU at 295 K, and 920 GHz/RIU at 300 K			
High-Q toroidal dipole resonance is based distorting symmetry in the bound state in the continuum	489 GHz/RIU		Modelling	[141] Wang et al. (2021)
	Q factor 1.2 × 10^5^			
Shift and magnitude of EIT resonances	496.01 GHz/RIU	Sensing glioma cells (mutant and wild) cultured on metamaterial surface	Polarization independent due to rotational symmetric design	[146] Zhang et al. (2021)
Two resonant absorption frequencies at 0.286 THz and 0.850 THz to observe frequency shift	29 and 74 GHz/RIU	Cervical cancerous tissues	Higher accuracy with 2 resonances than single	[158] Li et al. (2021)
Chiral sensing using chiral metasurface and qualitative identification of their D- and L-enantiomers	1×10^−5^ g/mL	Amino acid aqueous solution	//	[149] Zhang et al. (2021)
Single metallic bar array metamaterial distinguished by the resonance peak shifts	1 nM limit	Amyloid β peptide aggregates in a buffer solution detection	//	[148] Tang et al. (2021)

### 4.3 Quantum electronics

**Figure 16:**
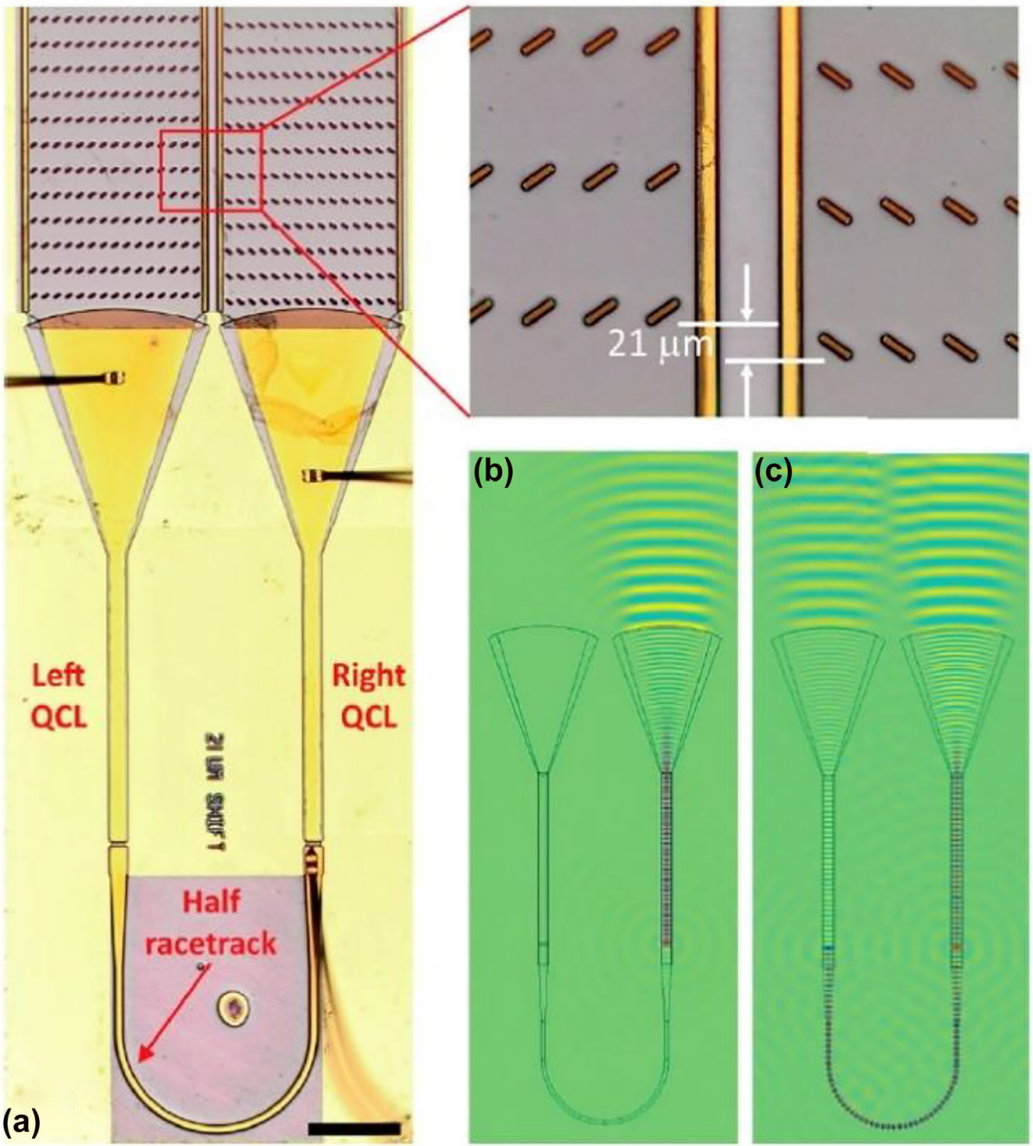
Integrated photonic metasurfaces for dynamic polarization tuning of QCLs’ emission from linear to circular, reproduced with permission from G. Liang et al., ACS Photon. 4: 517–524. Copyright (2017) American Chemical Society, Ref. [151].

**Figure 17:**
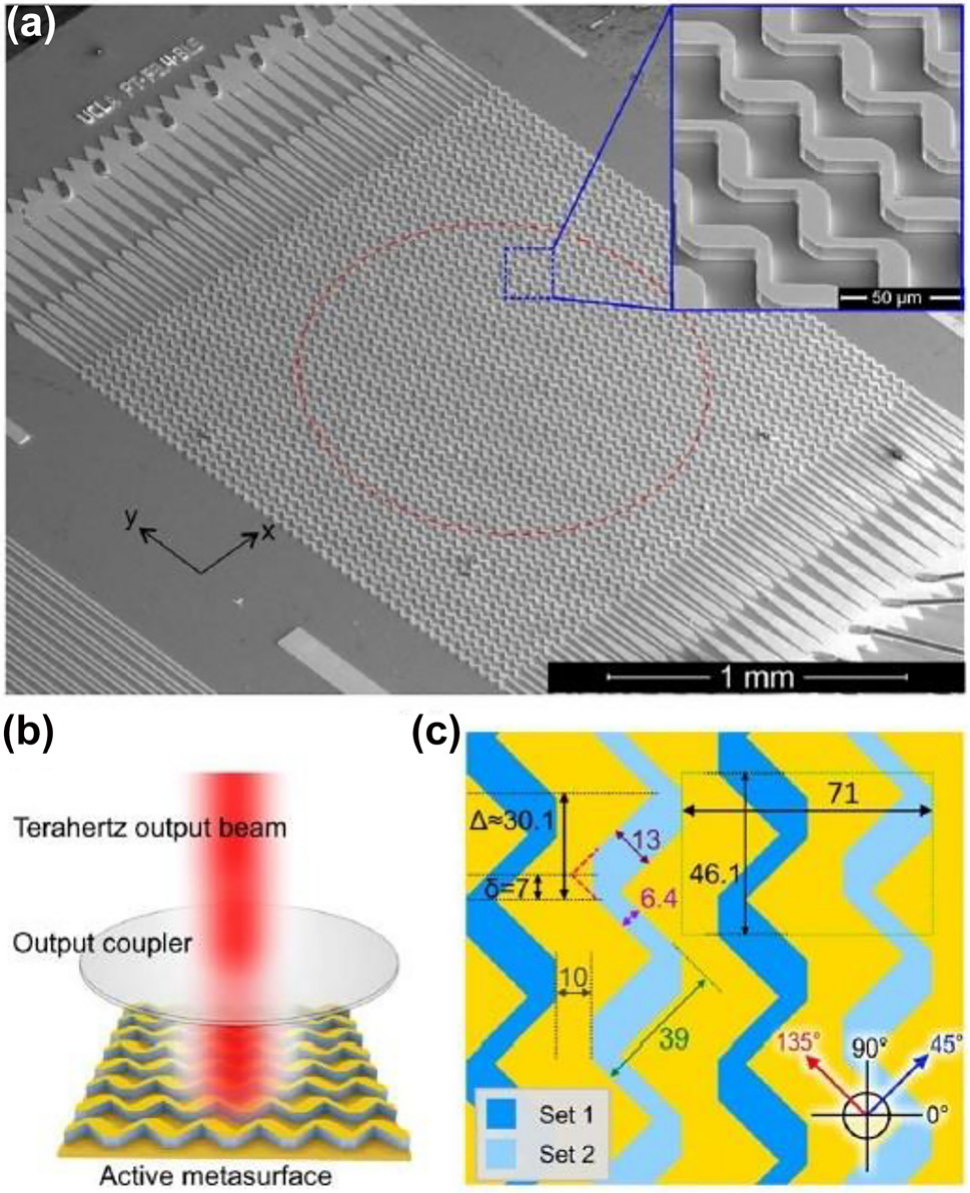
Cross oriented independently tuneable metasurfaces fabricated in the active region of a THz QCL in an external cavity arrangement. By independently biasing the alternate sets of zig-zag lines, a polarization control of 80° was achieved. Reprinted with permission from [153], © The Optical Society.

The introduction of terahertz metamaterial in quantum electronics concerns laser sources [149], [150], [151], [152], [153], emitters [156], [157], [158], [159], detectors [160] and unconventional quantum devices [161], [162], [163]. A semiconductor laser source typically covering the spectral region above 1.5 THz and below 5 THz is the THz quantum cascade laser. This unipolar semiconductor laser source is typically based on the repetition of a module comprising multiple quantum wells and barriers, engineered such to have transitions between minibands in the conduction band. The QCLs are ultrastable, narrow frequency sources emitting W-power levels and finding applications in many research fields, e.g., imaging and spectroscopy, but traditionally require cryogenic operation. Recently a breakthrough in QCL research has removed the main bottleneck preventing the diffusion of these sources beyond academia [164], reaching operating temperature of 250 K, thus allowing the use of thermoelectric cooling elements. Engineering these sources has always proved to be complex. The QCL frequency modulation achievable by directly acting on their driving current is normally limited to ∼10 GHz when feasible. Intersubband transition rules hinder a polarization emission other than TM mode. Because of the commonly used double metal laser cavity, the optical mode emitted is far from being Gaussian. Metamaterial modulators either integrated in the laser or in external cavity arrangement have demonstrated to be extremely effective in achieving polarization, frequency and amplitude modulation. An interesting approach [151], which allowed to overcome the TM mode QCL laser emission, consisted in the integration with two QCLs of two independently electrically tuneable metasurfaces, as showed in Figure 16. The two QCLs can be independently biased as well as the half racetrack, physically separated by an etched trench, needed to phase lock the two lasers. The metasurfaces are realised as metallic dipoles etched on a pedestal, tilted 45° with respect to the QCL cavity direction and cross- oriented. An elliptical polarization with about 630° optical activity was achieved with this arrangement for radiation emitted vertically, by independently acting on a single QCL per time. Linear to nearly circular polarization conversion was recorded when operating both QCLs at the same time. Alternatively, QCLs metamaterial photonic structures can be engineered directly in the active region of the material and together with an external coupler form a laser cavity, as showed in Figure 17, from [153]. The metasurface consists of two independently biased zig-zag patch antenna patterns. By alternatively biasing one set instead of the other, it was possible to select one polarization state at 45° instead of the other at 135° theoretically. This all-electronic tuneable bistability, allowed to achieve an 80° different polarization state without affecting the power, emission temperature and beam quality. The approach is based on the design of low Q-factor metasurfaces. The system required an external coupler to achieve lasing. The external coupler can be polarization/frequency insensitive, or it can be implemented as an etalon to reinforce frequency tuning or selection [154]. Alternatively, hybrid metamaterial/graphene frequency dispersive modulators were used as active external mirrors [152] in combination with QCLs. By changing the metasurface reflectance, via electrostatic graphene tuning, it was possible to modify the QCL spectral content all-electronically, without using any moving element. Further to this, amplitude stabilization of QCL was demonstrated by using a classic metamaterial arrangement based on SRRs and graphene, as external modulator [155]. All these approaches aimed to introduce a further versatile, efficient and, in most of the case, all-electronic modulation of the QCL emission by adding novel functionalities, or by providing other independent channels to control amplitude and frequency, normally strongly interconnected in these devices.

**Figure 18:**
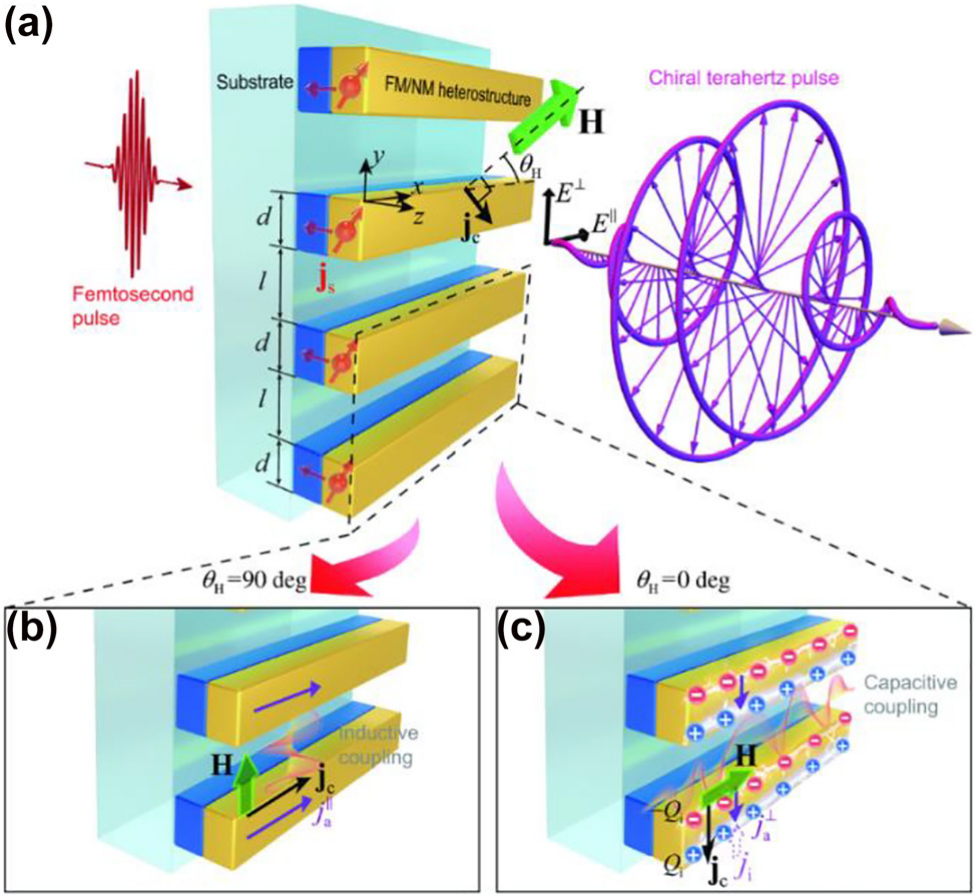
A chiral THz spintronic devices, reproduced from C. Liu et al. Adv. Photon. 3(5), 056002 (2021), Ref. [159].

**Figure 19:**
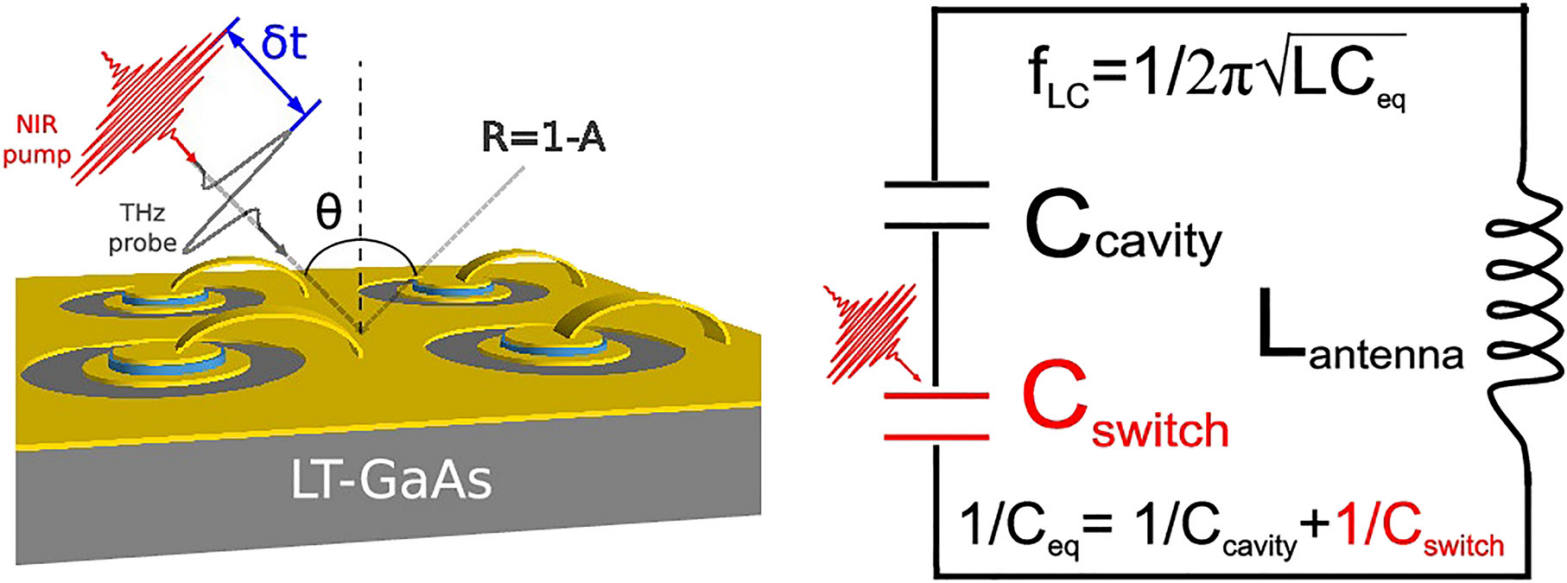
Ultrafast near infrared all optical switch of the LC resonance supported by 3D meta atoms, reprinted with permission from P. Goulain et al. ACS Photon. 8, 1097–1102. Copyright (2021), American Chemical Society, ref. [163].

Lately, a great attention has been placed on the development of spintronic emitters [158] for broadband THz generation in time-domain resolved spectroscopy upon illumination with high energetic near infrared fs pulses. The ultrafast femtosecond spin dynamics are responsible for the radiated THz radiation, and represent a valid alternative to the classical induced photocurrent in LT-GaAS based antennas in an Auston switch approach. The introduction of metamaterials was proposed and realised directly in the NIR thus enhancing the nonlinearity and efficiency of the incoming fs pulses [156]. However, a direct implementation in the THz output antennas would allow a direct tailored control of the emitted amplitude and phase. Such as arrangement was realised e.g., in [157], where the spintronics emitter was a W/CoFeB/Pt trilayer with an EIT metamaterial array integrated. The metamaterial structure based on EIT is inherently dispersive and can be modified in order to match directly the output phase and amplitude and thus achieve a tailored THz emission. However, metamaterial can be engineered directly into the spintronic material itself, as in [159], where the capacitive and inductive interplay between ferromagnetic and non-magnetic heterostructures, yielded an elliptical THz polarization output with >0.75 ellipticity over a broad (1–5 THz) range, as showed in Figure 18. The THz spintronic emission modulation is achieved either via lithography tuning, or by acting on the relative incident angle of the infrared fs pulses, but it is envisaged that more efficient modulation techniques will be implemented in the next future. THz meta-atoms have been investigated for the design of novel quantum detectors, e.g., in [160], and extensively studied for the realization of exotic quantum phenomena by modulating the strong light–matter interaction [159, 160]. Strong light–matter interaction takes place when the Rabi frequency, the energy exchange rate between light and matter, exceed the optical carrier frequency. An ultrafast, sub-cycle modulation of the metamaterial resonant cavity coupled to an electronic transition is therefore needed. A common approach based on the Landau level resonances, is compatible with standard planar metasurfaces, but it requires the use of a magnetic field and cryogenic temperatures. The engineering of multiple quantum wells coupling to TM polarization removes the need of a magnetic field, but requires the fabrication of an inherent 3D resonator [163]. This ultrafast all optical THz modulator is based on a 3D meta-structure, as shown in Figure 19, fabricated onto an LT-GaAs substrate. The LC resonance can be tuned by optically exciting the capacitance in the outer ring of the structure including LT-GaAs, whilst the capacity of the inner ring and the antenna’s inductance are not changed by the incoming fs pulses. The overall modulated reflectance is ∼2.2 ps by 280 GHz around 2–2.5 THz. The combination of a similar approach with the electronic transitions supported by multiple quantum wells is promising for the investigation of sub-cycle switching of the ultra-strong light matter regime in this frequency range.

### 4.4 Future perspectives

Motivated by the rapid progress made in the area of THz metamaterial modulators in the last few years, this review article intends to highlight the key developments recently made. Whilst it is difficult to predict how all the different methodologies will eventually evolve, it is still possible to identify some common trends going forward. From a technological point of view, the next generation of THz metamaterial modulators will focus on further integration of different functionalities, such as active beam forming and steering, real-time controllable phase gradients or distributions, coupled with a capability to engineer active wavefront control. Coded metamaterials represent another promising avenue that will continually attract attention for the implementation of logic gates, and programmable metasurfaces, where every single pixel will be independently addressable for holographic and communication applications. Indeed, next generation wireless communication is driving research in this field [165] highlighting the consideration of additional features such as frequency scalability, GHz-reconfiguration speed, all-electronic control, insertion losses, miniaturization, power consumption, ease of fabrication and robustness. All these factors point towards CMOS, semiconductors and graphene approaches. At the same time, there is a vibrant research taking place aiming at the implementation of new functional materials into active metasurfaces for fundamental research and ultrafast modulation. Amongst these materials include perovskites and 3D Dirac materials [166], which exhibits a linear gapless dispersion similar to graphene and Weyl semimetals [167, 168]. These materials have gained a wide popularity in recent years because of their outstanding optoelectronic properties such as ultrahigh electron mobility (∼7200 cm^2^ V^−1^ s^−1^ [169]), saturable absorption and short carrier recombination lifetime (in the range of ps), holding great promise for future modulation applications and thereof.

## 5 Conclusions

This review has comprehensively highlighted key progresses made in the fast-evolving field of terahertz modulation by using metamaterials in their broad definition, crystallizing the research picture that has been developing in these very recent years. The principal approaches, such as optical pumping, electrostatic gating and thermal tuning were critically considered. A few diverse active materials, e.g., semiconductors, superconductors, 2D and phase change materials, were also surveyed. In order to provide a direct comparison for the benefit of the scientific community, the performances of the different metamaterial modulators in terms of the established FOMs were tabulated. Finally, we also analysed the impact of this research in key strategic sectors, such as sensing, wireless communications and quantum electronics.
